# Mechanical stress induces a scalable switch in cortical flow polarization during cytokinesis

**DOI:** 10.1242/jcs.231357

**Published:** 2019-10-01

**Authors:** Deepika Singh, Devang Odedra, Priyanka Dutta, Christian Pohl

**Affiliations:** Buchmann Institute for Molecular Life Sciences and Institute of Biochemistry II, Medical Faculty, Goethe University, Max-von-Laue-Strasse 15, 60438 Frankfurt (Main), Germany

**Keywords:** Actomyosin, Cortical flow, Embryo, Cytokinesis, Mechanosensitivity, *C*. *elegans*

## Abstract

During animal development, cells need to sense and adapt to mechanical forces from their environment. Ultimately, these forces are transduced through the actomyosin cortex. How the cortex simultaneously responds to and creates forces during cytokinesis is not well understood. Here we show that, under mechanical stress, cortical actomyosin flow can switch polarization during cytokinesis in the *C. elegans* embryo. In unstressed embryos, longitudinal cortical flow contributes to contractile ring formation, while rotational cortical flow is additionally induced in uniaxially loaded embryos, i.e. embryos compressed between two plates. Rotational flow depends on astral microtubule signals and is required for the redistribution of the actomyosin cortex in loaded embryos. Rupture of longitudinally aligned cortical fibers during cortex rotation releases tension, initiates orthogonal longitudinal flow and, thereby, contributes to furrowing in loaded embryos. Moreover, actomyosin regulators involved in RhoA regulation, cortical polarity and chirality are all required for rotational flow, and become essential for cytokinesis under mechanical stress. In sum, our findings extend the current framework of mechanical stress response during cell division and show scaling of orthogonal cortical flows to the amount of mechanical stress.

## Abstract (precis)

**Summary:** When the *C. elegans* embryo experiences mechanical stress during its first cell division, the direction of cortical flow needs to change so cytokinesis can be completed.

## INTRODUCTION

While cells remodel their actomyosin cortex during cell division, they have to simultaneously integrate chemical and mechanical stimuli from the local environment to ensure successful cytokinesis. For cytokinesis to be robust – yet responsive – to extrinsic stimuli, three fundamental control principles have evolved, (a) redundancy (Srivastava et al., 2016), (b) mechanosensitivity (West-Foyle and Robinson, 2012) and, (c) positive feedback (Mandato et al., 2000). Examples for these control principles are (a) partially redundant actin crosslinkers and membrane trafficking pathways; (b) molecular mechanosensitivity of non-muscle myosin II (NMY-2), α-actinin and filamin (Luo et al., 2013; Schiffhauer et al., 2016) and; (c) RhoA-dependent self-enhancing local assembly and contraction of actomyosin, as well as astral microtubule-based suppression of actomyosin contractility (Mangal et al., 2018), which both are required to generate cortical contractile actomyosin flow during cell division.

Work in the last decade has led to the identification of the main mechanosensory system that operates during cell division. The core of this system is NMY-2, which amplifies sensed forces through its lever arm (Luo et al., 2013), and which shows mechanosensitive accumulation through cooperative binding to F-actin (Luo et al., 2012). This results in positive feedback on the assembly of NMY-2 bipolar thick filaments (West-Foyle and Robinson, 2012).

Among the control principles mentioned above, feedback during cytokinesis crucially depends on spindle microtubules since they constitute key modulators of cortical contractility (Mandato et al., 2000). Lewis Wolpert initially proposed the astral relaxation model, in which astral microtubules soften the polar cortex (by suppressing actomyosin contractility), while the equatorial cortex stiffens during division (Wolpert, 1960). Very recently, it has been shown that polar clearing of contractile ring components requires TPXL-1-dependent cortical activation of Aurora A (Mangal et al., 2018), confirming parts of the astral relaxation model.

It has also been shown that cortical flow leads to contractile ring formation by alignment of actin filaments in the *C. elegans* one-cell embryo due to compression of the gel-like cortex in the equatorial region (Reymann et al., 2016). Moreover, it has been suggested that a positive feedback between cortical myosin of the contractile ring and flow of cortex into the ring gives rise to an increase in contractile ring myosin to maintain a high ring constriction rate (Khaliullin et al., 2018). These findings suggest that NMY-2-dependent flow can re-organize the cortical actin network during cytokinesis – as has been proposed previously (Bray and White, 1988).

Cortical contractile actomyosin flows in the *C. elegans* embryo are strictly dependent on RhoA activation and do not only cause translation of the cortex – like during anteroposterior polarization (Munro et al., 2004) – but also rotation immediately before division of the two-cell embryo (Schonegg et al., 2014; Singh and Pohl, 2014), and during chiral symmetry breaking (Naganathan et al., 2014; Pohl, 2015). Cortex rotation occurs during cell division when cytokinetic actomyosin foci have formed. This mesoscopic rotational flow is most likely due to generation of torque at the molecular level. It has previously been shown *in vitro* that torque is generated during myosin-driven sliding of actin filaments (Nishizaka et al., 1993), which induces a right-handed rotation of an actin filament around its long axis with one revolution per sliding distance of ∼1 μm (Sase et al., 1997). Similar rotation or twirling of actin filaments have been confirmed in more-recent reports (Beausang et al., 2008; Vilfan, 2009). Although the molecular origin of torque in actomyosin dynamics is well understood, how torque leads to coordinated cortical rotational dynamics remains unexplored. Moreover, not only the cortex of the *C. elegans* one-cell embryo seems to rotate but, most likely, the entire spindle and the nuclei (Schonegg et al., 2014; Bolková and Lanctôt, 2016). Thus, macromolecular torque on the cortex might be intricately linked to spindle dynamics, especially since cortical torque has so far only been described during polarization and cell division (Naganathan et al., 2014; Singh and Pohl, 2014), both processes that require dynamic microtubule activity.

Previously, it has been shown through highly informative ablation experiments of the contractile ring that it is able to repair gaps in itself requiring an increased tension in the ring and reduced cortical tension in the vicinity (Silva et al., 2016). The former is mediated by recruitment of new material and actin polymerization, the latter most likely by disassembly of contractile elements outside of the equatorial zone of activated RhoA. This suggests that global cortical dynamics respond to mechanical stress during cytokinesis, which might require differential regulation of cytokinetic cortical flow.

Here, we quantitatively describe the biomechanical responses to mechanical stress through loading. For this, progressive uniaxial loading, i.e. pressure, was used in the form of the parallel plate assay (Cole, 1932; Yoneda and Dan, 1972; Fischer-Friedrich et al., 2014). In this assay, two parallel plates are used to deform an object – in our case the *C. elegans* cortex – by compressing it between them. With this simple mechanical manipulation, it is possible to demonstrate that a recently uncovered type of polarizing cortical flow, i.e. rotational flow (Naganathan et al., 2014; Schonegg et al., 2014; Singh and Pohl, 2014), is mechanoresponsive and scales to the amount of load, thereby contributing to successful division when cells experience mechanical stress. Anisotropic accumulation of NMY-2 suggests that cortical stress is similarly anisotropic in uniaxially loaded embryos, as has recently been shown for uniaxially loaded mammalian cells (Fischer-Friedrich et al., 2016). Importantly, rotational flow leads to a re-arrangement of the anisotropically distributed actomyosin in loaded embryos. Cortical rotation requires a broad set of actomyosin regulators, of which several only become essential for cytokinesis under mechanical stress. We also demonstrate that rotational dynamics seem to emanate from the mitotic spindle, whereby astral relaxation appears to be the main driving force of furrow-directed cortical flow. Hence, our data suggest that the main biological role of cortical flow re-polarization during cytokinesis lies in balancing spatial and tension anisotropies in the cortex, and that a converging longitudinal flow is required for successful furrowing in mechanically stressed embryos.

## RESULTS

### Convergent longitudinal flow polarizes cortical NMY-2

**Fig. 1.**
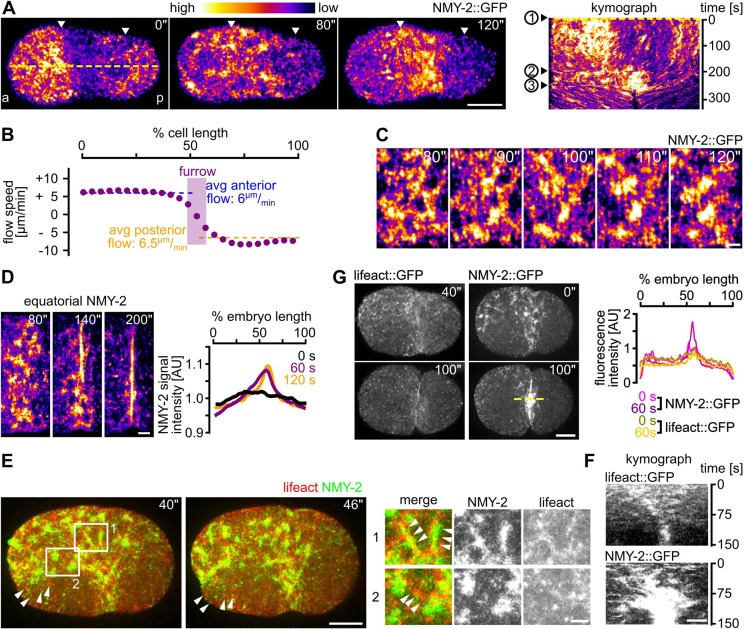
**Longitudinal flow organizes cortical NMY-2 during the formation of the contractile ring.** (A) Left: Maximum projected stills from time-lapse microscopy of embryos expressing NMY-2::GFP. White arrowheads mark the boundaries of the anterior (a) and posterior (p) NMY-2 caps upon polarization. Right: Kymograph along the yellow dashed line shown in the leftmost panel. Numbers on the left refer to onset of cap formation (1), onset of NMY-2 cytokinetic foci formation (2) and start of furrow invagination (3). Scale bar: 10 µm. See also Movie 1. (B) Average cortical NMY-2 flow velocity profile along the anterior–posterior axis generated from PIV data of five embryos during the time window of longitudinal flow (60 s). (C) Maximum projected stills from time-lapse microscopy of the furrow region; scale bar: 2.5 µm. (D) Left: Stills from maximum projected embryos showing NMY-2 dynamics at the equatorial ring. Scale bar: 2.5 µm. Right: Normalized NMY-2::GFP signal intensities along the anterior–posterior axis in one-cell embryos. Intensity profiles at 0 s, 60 s and 120 s are represented by black, purple and yellow traces, respectively (*n*=5 each). (E) Left: Organization of NMY-2 and F-actin during onset of cytokinesis. Maximum projected stills from time-lapse microscopy of embryos expressing lifeact::mCherry (red) and NMY-2::GFP (green). White arrowheads mark persistent actin foci. Scale bar: 10 µm. Magnifications of boxed areas 1 and 2 are shown in right panel. Right: Localization of NMY-2 on actin filaments (1) and NMY-2 foci connected by actin filaments (2). Scale bar: 2.5 µm. (F) Kymographs for lifeact (top) and NMY-2 (bottom) at the midbody region (generated along the dashed yellow line shown in G. Scale bars: 2.5 µm. (G) Left: Maximum projected stills from time-lapse microscopy of representative wt embryos expressing either lifeact::mCherry or NMY-2::GFP. The midbody region is indicated by a dashed yellow line. Scale bar: 10 µm. Right: Quantification of signal intensities from embryos depicted in the left panel. Time stamps in all panels refer to 0 s= formation of the polar cap at onset of cytokinesis.

To establish an unbiased readout for cortical dynamics during cytokinesis, we performed time-lapse microscopy of the first division in wild-type (wt) *C. elegans* embryos expressing the CRISPR/Cas9-edited NMY-2 fused to GFP (NMY-2::GFP; Dickinson et al., 2013). These data (Fig. 1A) were then subjected to quantitative analysis by particle image velocimetry (PIV). PIV revealed longitudinal cortical NMY-2 flows with opposite direction, from anterior (6±0.05 µm/min, *n*=5) and posterior poles (6.5±0.09 µm/min, *n*=5) towards the cell equator (Fig. 1B, top panel; Movie 1). Convergence of flows at the equator leads to the transformation of cortical NMY-2 foci (1.8±0.1 µm in diameter, *n*=25) into parallel, linearly organized NMY-2 (0.25–0.5 µm in width and 3.5±0.6 µm in length, *n*=20; Fig. 1A,C) that first form a narrow strip (6.8±0.07 µm; *n*=5) and, subsequently, become part of the incipient contractile ring by alignment and bundling (Fig. 1D; Movie 1). Importantly, not all foci transform into linearly organized NMY-2; many dissociate prior to or during transformation since they have a limited lifetime (see below).

Previously, a physical model based on hydrodynamic active gel theory has explained formation of the F-actin component of the contractile ring by cortical flow (Salbreux et al., 2009; Reymann et al., 2016). In this model, opposing flows that emerge at the poles, converge at the equator to promote ordering of cortical actin filaments into parallel bundles (Fig. S1A). Our analyses revealed similar ordering for myosin during furrow-directed cortical flow (Fig. S1B). Moreover, these dynamics are highly consistent with the recently proposed constriction-coupled disassembly and compression feedback regulation for myosin at the equator ring during cytokinesis (Khaliullin et al., 2018). Here, cortical flow transports cortical material to the equatorial zone of activated Rho signaling, where myosin can accumulate and trigger cortex compression by recruiting the adjacent cortex, and assist in actin depolymerization (Fig. S1C).

Analysis of NMY-2 foci dynamics during longitudinal flow revealed an average lifetime of 29±2 s (*n*=25), whereas analysis of F-actin (using lifeact::mCherry; Pohl et al., 2012) showed a distribution into two populations; one filamentous with a smooth texture and a short lifetime, and another that does not concentrate in cortical NMY-2 foci and forms much smaller, uniformly sized (0.4±0.1 µm; *n*=20) and long-lived (124±48 s; *n*=20) foci that do not undergo changes during cytokinesis (Fig. 1E, arrows). Nevertheless, NMY-2 decorates smooth filamentous actin shortly after onset of cytokinesis (Fig. 1E), whereas actin filaments subsequently disassemble after ∼15 s (based on lifeact turnover measurements), linearly organized NMY-2 and NMY-2 foci persist substantially longer (Fig. 1F). Consistent with smooth F-actin showing faster cortical turnover, we also found that F-actin shows slightly weaker longitudinal flow with a shorter range (0.3±0.2 embryo lengths) when compared to NMY-2 (0.6±0.1 embryo lengths). This difference can also be explained by the constriction-coupled disassembly and compression feedback regulation, where actin is disassembled during cortical compression at the equator while myosin keeps accumulating (Fig. 1G). This is most striking during late cytokinesis, when substantial amounts of linearly organized NMY-2 still flow towards the future midbody, while F-actin does not show any recognizable flow at that stage (Fig. 1F). These observations also suggest – similar to what has been recently found in mammalian tissue culture (Hu et al., 2017) – that NMY-2 organizes in aligned stacks within the *C. elegans* cortex, which can span several micrometers and whose turnover is independent of the turnover of actin filaments.

### Uniaxial loading counteracts longitudinal flows

**Fig. 2.**
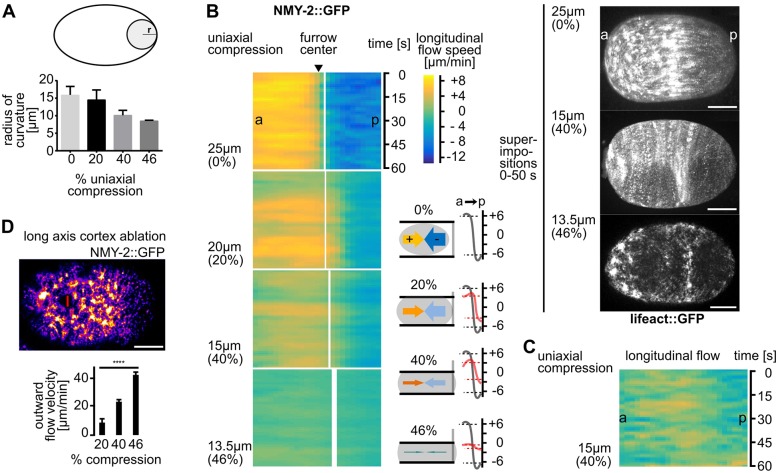
**Longitudinal NMY-2 flow is mechanosensitive.** (A) Quantification of curvature increase due to compression. Smaller radii represent higher curvature (see cartoon at top, and Materials and Methods). (B) Left: Heat map kymographs of cortical flow velocities obtained from PIV of NMY-2::GFP foci moving along the long axis of differently mounted one-cell *C. elegans* embryos. For statistical parameters of heat maps see Fig. S1A. Black arrowhead points to the white line demarcating the future furrow; thickness of the line represents standard deviation; a, anterior; p, posterior. Bottom middle: Schematic highlighting the paradigm of uniaxial compression and corresponding flow velocities. Bottom right: Averaged velocities (over 60 s) along the anterior–posterior axis (a→p) from the PIV analysis (right panels). Grey and red curves represent averaged velocities in uncompressed and compressed embryos, respectively (*n*=5 each). Right: Superimpositions generated by overlaying stills from projected time-lapse images. Scale bars: 10 µm. (C) Heat map kymographs generated by PIV of lifeact::mCherry for longitudinal flow. Embryos were imaged under 40% compression (*n*=5). (D) Top: Representative still of an NMY-2::GFP-expressing embryo exhibiting a cortical wound inflicted by UV laser cutting along the short axis of the embryo. Bottom: Quantification of outward flow velocities following cortical wounding under increasing compression (*n*=5 each). See also Movie 3.

To probe cytokinesis mechanics, we used the well-established parallel plate assay (Cole, 1932; Yoneda and Dan, 1972; Fischer-Friedrich et al., 2014). To achieve highly consistent uniaxial loading in the parallel plate assay, we employed monodisperse, inert beads with diameters of 25, 20, 15 and 13.5 µm, (representing 0, 20, 40 and 46% of uniaxial compression, respectively; Movie 2). Loading induces shape anisotropy, where surfaces in contact with the plates become flat and the remaining surfaces start bulging. It has been shown that uniaxial loading directly impinges on cortex mechanics because (a) the cell boundary is governed by Laplace's law (Fig. 2A; i.e. deformation leads to increased cortical tension as the large elastic modulus of the cytoplasm requires an immediate force balance at the cell boundary, which is proportional to the change in the curvature; Fischer-Friedrich et al., 2014); (b) external friction (friction between the actomyosin cortex and the egg shell) can be neglected (Mayer et al., 2010; Turlier et al., 2014); (c) the elastic cortical layer dominates mechanics in the system, whereas the plasma membrane can be largely ignored (Tinevez et al., 2009; Turlier et al., 2014; Fischer-Friedrich et al., 2016). By analyzing longitudinal NMY-2 cortical flow prior to furrowing, we found that longitudinal flow velocities are highest in unloaded embryos and decrease with increased loading (Fig. 2B, left). Flow velocities were down to 3.5 and 3.4 µm/min in anterior and posterior domains, respectively, in embryos compressed 20%, and decreased further to 1.8 and 2.8 µm/min at 40% compression (Fig. 2B; Fig. S2A). However, wt embryos compressed 46% reach only −0.7 and 1.8 µm/min (anterior and posterior, respectively) and fail to cleave (Movie 2). The strong reduction of longitudinal flow, i.e. flow along the anterior–posterior axis, is most apparent in superimpositions of consecutive frames from time-lapse recordings (Fig. 2B, right). Interestingly, the reduction of longitudinal flow scaled to the amount of loading, suggesting that the cortex behaves like a Newtonian material within this range of stress (Fig. S2B).

Consistent with F-actin showing faster cortical turnover, we found slightly weaker and less uniform longitudinal flow for F-actin (lifeact) compared to NMY-2 (Fig. 2C, Fig. S2A). Since uniaxial compression induces a shape anisotropy that leads to anisotropic stress in the cortex (Fischer-Friedrich et al., 2016), this might alter cortical tension and impinge on longitudinal cortical flows. To test this, we performed cortical laser ablations just prior to the onset of polarizing flow after fertilization parallel to the short axis of the embryo (cuts of 23% embryo width; Fig. 2D, Movie 3). We chose this time point for ablations since the cortex shows a highly similar architecture to the cortex just prior to cytokinesis (Reymann et al., 2016) and the measurements are not confounded by fast-changing patterns of flows. We ensured that cortical wounds did not vary in size at different degrees of compression (Fig. S2C). Measuring outward velocities of NMY-2 foci post ablation, we found that increased loading generates increased outward flow velocities (11±0.6 µm/min at 20% compression, 23±1 µm/min at 40% compression, and 43±2 µm/min at 46% compression; Fig. 2D). Although our ablation experiments were performed before onset of cytokinetic flows, they clearly demonstrate a response of the cortex that, nevertheless, scaled to loading. Thus, our observations are consistent with uniaxial compression inducing cortical stress that seems to counteract longitudinal flow (Fig. 2B), eventually preventing successful furrowing.

### Rotational flow is induced upon uniaxial loading

**Fig. 3.**
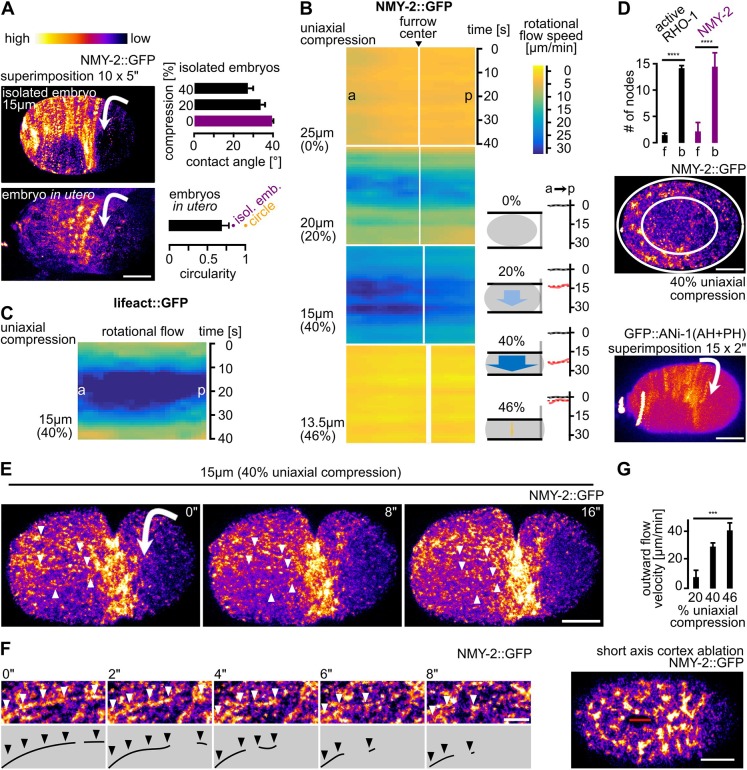
**Rotational cortical flow is required for furrowing under uniaxial compression.** (A) Left: Maximum projected stills from time-lapse microscopy of a representative isolated wt embryo (top) and an embryo inside the uterus (bottom; see also Fig. S3; Movie 4); scale bar: 10 µm. The direction of cortical rotation is indicated by arrows. Top right: Contact angles between coverslip and embryo. Bottom right: Circularity of embryos *in utero* (*n*=6), circularity for ellipsoidal isolated embryos and a circle are also included. (B) Left: Heat map kymographs of cortical flow velocity values from NMY-2::GFP particle tracking along the short axis of differently mounted embryos. For statistical parameters of heat maps see Fig. S2D. Black arrowhead points to the future furrow center; a, anterior; p, posterior. Middle: Schematic highlighting the corresponding rotational cortical flow velocities. Right: Averaged velocities (over 60 s) along the anterior–posterior axis from the PIV analysis (left panels). Grey and red curves represent averaged velocities in uncompressed and compressed embryos, respectively (*n*=5 each). (C) Heat map kymographs generated by PIV of lifeact::mCherry for rotational flow. Embryos were imaged under 40% compression (*n*=5). (D) Top: Quantification of active RHO-1 (black) and NMY-2 (purple) foci on flat (f) versus bulging (b) surfaces in embryos under 40% compression (*n*=5 each). Middle: Representative projection of an embryo illustrating the quantification for NMY-2::GFP; inner ellipse=flattened surface (see E for fluorescence intensity color code). Bottom: Superimposition of frames from a representative embryo expressing a sensor for active RhoA, GFP::ANI-1(AH+PH). Note that active RhoA is also found outside the equatorial zone. Arrow indicates direction of rotation, see also Movie 6. Scale bar: 10 µm. (E) Projections from time-lapse data (see Movie 5). Arrowheads point to linear cortical NMY-2 that undergoes rupture. Scale bar: 10 µm. (F) Magnified projection of the cortex showing rupture of linearly organized NMY-2. Scale bar: 2.5 µm. (G) Top: Quantification of outward flow velocities following cortical wounding under increasing loads (*n*=5 each). Bottom: Representative still from a NMY-2::GFP-expressing embryo exhibiting a cortical wound inflicted along the long axis of the embryo by UV laser cutting. Scale bar: 10 µm. See also Movie 8. Time stamps in the panels arbitrarily refer to time points of 0 s.

Work by us and others has uncovered rotational flow of the cortex – orthogonal to longitudinal flow – in the one-cell *C. elegans* embryo directly before contractile ring formation (Fig. 3A, top left) (Schonegg et al., 2014; Singh and Pohl, 2014). This rotational flow also occurs *in utero* (Fig. 3A, left; Fig. S3; Movie 4) and is most likely due to deformations of embryos *in utero,* similar to 20–40% uniaxial loading of embryos when comparing contact angles (Fig. 3A, right). However, whether rotational flow is an intrinsic property or is induced has, so far, not been addressed. By utilizing the paradigm of uniaxial loading of the parallel plate assay, we observed that, whereas longitudinal NMY-2 flow velocities decrease, rotational cortical flow velocities increase concomitantly from 0.8±0.02 µm/min in uncompressed embryos to a maximum of 23±0.1 µm/min in embryos compressed 40% (Fig. 3B; Fig. S2D; Movies 2 and 5). Under very high loading, rotational flow is virtually absent due to accumulation of NMY-2, F-actin and activated RhoA on bulging surfaces (see below). This shows that rotational flow is most likely an induced flow strongly enhanced by mechanical stress. Again, consistent with F-actin showing faster cortical turnover, we also find that F-actin shows a shorter range of rotational flow (Fig. 3C, Fig. S2D). Importantly, the magnitude of rotational cortical flow scaled to the amount of loading (Fig. S2E). Together with the scaling of longitudinal flows (Fig. S2B), this suggests that the two phenomena are not simply coincide but, most likely, are interdependent.

On the basis of these findings we asked how stress created by uniaxial loading contributes to rotational flow. By analyzing the distribution of NMY-2, F-actin and active RHO-1 (using a RhoA sensor consisting of GFP fused to the AH- and PH-domains of ANI-1; Tse et al., 2012), we found cytokinetic foci assembling uniformly in uncompressed embryos. By contrast, in compressed embryos, NMY-2, F-actin and active RhoA were only found at the equator and on bulging surfaces (Fig. 3D; Movie 6). This suggests that cell cycle-dependent RhoA activation is local and, most likely, in response to cortical deformation. Shortly after their assembly, focally and linearly organized NMY-2 moved onto flattened surfaces through rotational flow (Fig. 3E; Movie 7). Owing to actomyosin being concentrated on bulging surfaces in loaded embryos, its mobilization by rotational flow generated a flow front – the former boundary between the bulged and flat cortex – that moved over the flattened surface until the front reached the bulged surface on the other side (Movie 7).

Moreover, linearly organized NMY-2 connecting cytokinetic foci ruptured in compressed embryos (Fig. 3E,F; Movie 7). Rupture occurred anisotropically in the direction of rotation, starting at the front of rotational flow (Movie 7). This always lead to asymmetric positioning of the midbody (*n*>20; data not shown). Additionally, rupture lead to both flow towards the furrow (from the furrow-facing side of the rupture) and flow towards the poles (from the pole-facing side of the rupture) (Movie 5). Moreover, rupture lead to both flow towards the furrow (from the furrow-facing side of the rupture) and flow towards the poles (from the pole-facing side of the rupture) (Movie 7). Flow towards the furrow had similar velocities as longitudinal flow in uncompressed embryos. Flow towards the poles dissipated due to dissolution of foci and lack of a barrier similar to the equatorial band of focal and linear NMY-2 (Movie 7). Furthermore, as flow occurred at the same time polar blebbing was observed, it might contribute to cortical relaxation of cortical tension caused by pole-directed cortical flow (Fig. S4A).

Since uniaxial compression leads to anisotropic cortex assembly at the onset of cytokinesis and anisotropic disassembly during furrowing, we asked whether loading induces anisotropies in cortical tension that also contribute to rotational flow. To test this, we performed laser cutting of the cortex (cuts of 16% embryo length; Fig. 3G; Movie 8) parallel to the long axis of the embryo, just prior to the onset of polarizing flow after fertilization and observed a loading-dependent increase in initial outward flow velocities of NMY-2 particles at the site of the cortical wound (15±0.5 µm/min at 20% compression, 29±2 µm/min at 40% compression, and 32±4 µm/min at 46% compression; Fig. 3G).

When measuring outward velocities 5 s after cortex ablation (as established previously; Mayer et al., 2010), it seemed that tension increases along the short axis, scaling more linearly with loading (Fig. 3G; R^2^=0.94; Fig. S2G) than along the long axis (Fig. 2D; R^2^=0.83; Fig. S2G). Also consistent with previous work (Mayer et al., 2010), tension seemed to be higher along the short axis under low loading.

### Uniaxial loading and the limit of cytokinetic mechanostability

Next, we asked how rotational flow changes when embryos are subjected to 46% compression, a load under which embryos do not divide (Movie 9). Here, we found the same anisotropic distribution of foci that was observed at 20% or 40% compression; however, foci on bulged surfaces did not translocate by rotational flow. Instead, NMY-2 tracking revealed shear flow of NMY-2 in the equatorial area (Movie 9). Shear flow did not lead to the bundling of linear NMY-2 at the equator and the equatorial band of NMY-2 disintegrates (100% of embryos; *n*>15). Moreover, under 46% compression, actomyosin recruitment to the equatorial zone by the central spindle pathway was still observed; however, equatorial actomyosin recruitment was insufficient for furrowing. Similar to human cells (Fischer-Friedrich et al., 2014), we found that the limit of cortex loading in *C*. *elegans* is reached at 52% (12 µm beads; 50% for human cells). Owing to increased bulging, the cortex ruptures at these bulged sites and the equatorial NMY-2 band disintegrates (Fig. S4B, Movie 10). This confirms that the cortex is bearing the load of compression since we neither observed rupture of the plasma membrane nor of the eggshell.

### Actomyosin regulators are required for mechanostable cytokinesis

**Fig. 4.**
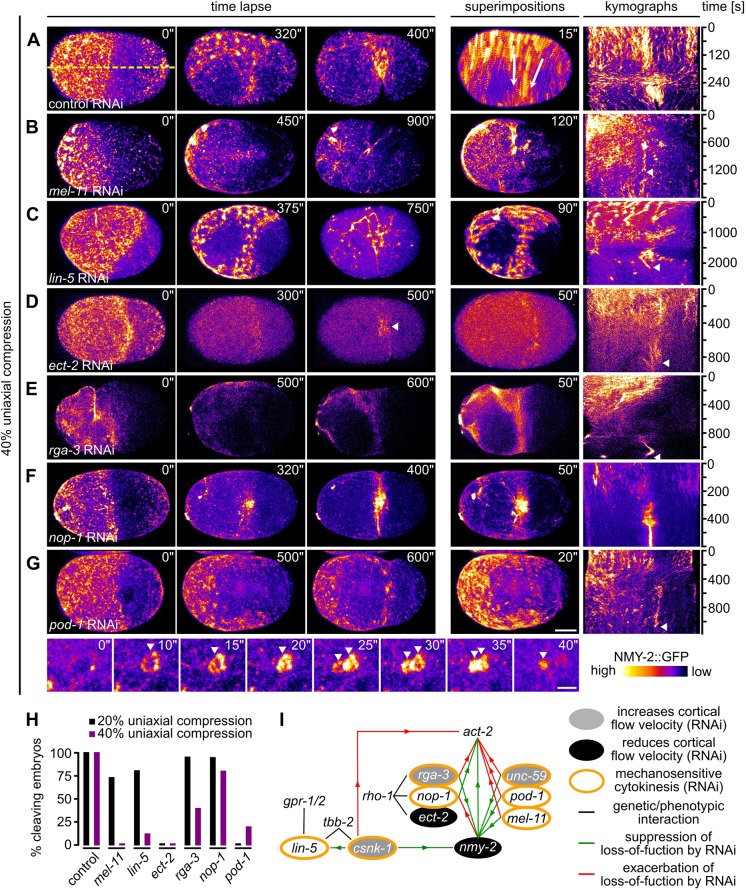
**Actomyosin regulators are required for rotational flow and cytokinesis mechanostability.** (A) Left: Maximum projected stills from time-lapse microscopy of a representative wt embryo expressing NMY-2::GFP. Middle: Superimposition of frames from a 15 s time window. White arrows indicate direction of rotational flow. Right: Kymograph generated along the dashed yellow line in the leftmost panel. (B–G) Maximum projected stills as in A, but for RNAi-treated embryos as indicated. All embryos were 40% compressed. Scale bar: 10 µm. See also Movies 11–16. Bottom panels of G: Magnification of projected stills showing formation of cortical circular structures (arrowheads) in *pod-1* RNAi embryos, here time of 0 s is arbitrary. Scale bar: 2.5 µm. See also Movie 16. (H) Quantification of successful first cell division for the indicated RNAi embryos under 20% (black) or 40% (purple) compression (*n*≥5 each). (I) Genetic network of factors controlling cytokinesis. Interactions are based on Fievet et al., 2013 and Naganathan et al., 2014 Yes, name should be 'Naganathan' throughout! (increase and/or decrease of cortical flow) and on our data shown in H (mechanosensitive cytokinesis).

Next, we performed a targeted screen to identify factors involved in cortical rotation and linear organization (Fig. S5). For the screen, we compressed embryos 20% or 40%, where strong rotational flow was observed in wt embryos (Fig. 4A). This screen identified six proteins for which cortical rotation is either lost or very strongly reduced, and for most of which cytokinesis is blocked at 40% compression (Fig. 4A–G). These include (1) MEL-11, a myosin-associated phosphatase (Piekny and Mains, 2002), required for both focal and linearly organized NMY-2 (Fig. 4B; Movie 11); (2) LIN-5, a protein known to regulate spindle positioning (Lorson et al., 2000), which also promotes the transition from focal to linear organization and seems to stabilize the latter (Fig. 4C; Movie 12); (3) ECT-2, a cytokinesis regulatory RhoGEF (Morita et al., 2005), which is required for the correct size and density of focal and linear NMY-2 (Fig. 4D; Movie 13); (4) RGA-3, a cytokinesis regulatory RhoGAP (Schonegg et al., 2007; Schmutz et al., 2007), whose depletion leads to exaggerated rotational flow (Dutta et al., 2015), and which we found to be required for foci formation and to suppress excess linear organization (Fig. 4E; Movie 14); (5) NOP-1, a factor required together with the RhoGAP CYK-4, in order to promote RHO-1 activation and NMY-2 foci formation during cytokinesis (Tse et al., 2012), and which is also required for the transition to linearly organized NMY-2 (Fig. 4F; Movie 15); (6) POD-1, a type III coronin implicated in actin dynamics and crosslinking (Chan et al., 2011), that is also required for this transition (Fig. 4G, top panels; Movie 16). Moreover, RNA interference (RNAi) of *pod-1* lead to the formation of short-lived circular contractile NMY-2 structures, which suggests that coronin-mediated actin crosslinking is required to coordinate formation of long-range NMY-2 linear organization to achieve pole-to-equator flow (Fig. 4G, bottom panels; Movie 16).

Although RNAi of these regulators is expected to alter cortical dynamics, for five factors, furrowing phenotypes have not been identified in previous studies (MEL-11, LIN-5, RGA-3, NOP-1 and POD-1). Their depletion lead to a loading-dependent failure of cytokinesis completion, meaning that the rate of successful cleavage reduced with increased loading (Fig. 4H). This was mirrored by a lack or strong reduction of rotational flow, partial phenotypes being due to incomplete penetrance of RNAi. Only for *ect-2* RNAi a complete block of cytokinesis is expected. Hence, in the aforementioned cases, cytokinesis occurred normally until the system encountered a threshold stress level. Here, *pod-1* RNAi was an exception, since increased loading lead to an amelioration of the phenotype when loading is increased. Remarkably, all regulators are known to have opposing effects in actin (*act-2*) and myosin (*nmy-2*) loss-of-function mutants in that they can suppress one mutant and exacerbate the phenotype in the other (Fievet et al., 2013; summarized in Fig. 4I). Moreover, they are either directly or indirectly linked to the Rho GTPase cycle (Fig. 4I). Hence, a network of factors required for coordinating balanced activation of actin and myosin is essential for cytokinesis' mechanical robustness.

### Persistent linearly polarized NMY-2 prevents cortical rotation

**Fig. 5.**
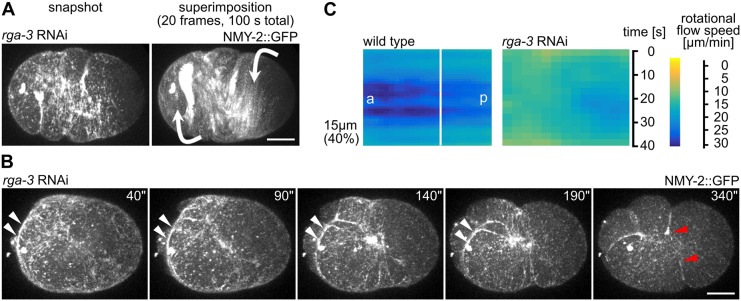
***rga-3* RNAi leads to increased linear organization of cortical NMY-2 and lack of rotational flow under load.** (A) Still and superimposed stills from time-lapse microscopy of a representative *rga-3* RNAi embryo. Note the linear organization of NMY-2 and the almost exclusive rotational trajectories of cortical NMY-2 in the superimposition. Direction of rotational trajectories (arrows) has opposite polarity (anterior domain counterclockwise, posterior domain clockwise). Scale bar: 10 µm. (B) Stills from a time-lapse series of a representative *rga-3* RNAi embryo during cytokinesis. White arrowheads mark long linear cortical NMY-2 that peels from the sides towards the nascent midbody. Red arrowheads mark the dissolving furrow. Scale bar: 10 µm. (C) Heat map kymographs of rotational cortical flow velocity values from NMY-2::GFP particle tracking along the short axis of wt and *rga-3* RNAi embryos, 40% uniaxial compression (*n* =5 each); a, anterior; p, posterior.

Previously, it has been shown that RNAi of *rga-3* leads to exaggerated chiral flows during anterior–posterior polarization of the one-cell *C. elegans* embryo (Naganathan et al., 2014; Dutta et al., 2015). However, the data above show that *rga-3* RNAi embryos do not divide under uniaxial compression. We, therefore, investigated more closely the origin of exaggerated chiral flows in *rga-3* RNAi embryos and why this prevents cytokinesis under mechanical stress. Although we observed the reported exaggerated chiral flow during anterior–posterior polarization under uniaxial loading (Fig. 5A), an important additional phenotype of *rga-3* RNAi embryos was the increased linear organization of cortical NMY-2, which can be observed both during anterior–posterior polarization (Fig. 5A) and right after the onset of cytokinesis (Fig. 5B). This organization was maintained during cytokinesis and lead to peeling of the filaments towards the nascent midbody, lack of a correctly formed contractile ring (Fig. 5B), and substantially reduced rotational flow under load (Fig. 5C). Thus, unlike in wt embryos, linearly organized cortical NMY-2 does not rupture in *rga-3* RNAi embryos. Considering the theory of cortical torques (Naganathan et al., 2014) it seems that a persistent linear organization of NMY-2, as that seen in in *rga-3* RNAi embryos, can induce stronger and more long-ranged torques than those seen in wt embryos, which seems to result in counter-rotating flows on the two sides of the furrow (Fig. 5A, white arrows). We propose that this interrupts cytokinesis since long-range linear connections cannot be remodeled (Fig. 5B).

### Astral relaxation drives cortical flow and is required for cortical rotation

It has recently been suggested that the whole embryo – in particular the spindle – rotates during the first cell division (Schonegg et al., 2014; Bolková and Lanctôt, 2016). We, therefore, wanted to test whether spindle-based mechanisms are responsible for cortical flow polarization and re-polarization under load. According to the astral relaxation theory (Wolpert, 1960), a gradient of contractility from the poles to the equator is sufficient to induce furrowing (Yoneda and Dan, 1972; Bray and White, 1988; Turlier et al., 2014). Contacts between the cortex and the astral microtubules of the spindle contribute to the formation of this gradient (Wolpert, 1960; Tse et al., 2011). Furthermore, the aster-based inhibitory signal leading to the clearing of the polar cortex from components of the cytokinetic furrow is Aurora A-mediated activation of TPXL-1 (Mangal et al., 2018).

**Fig. 6.**
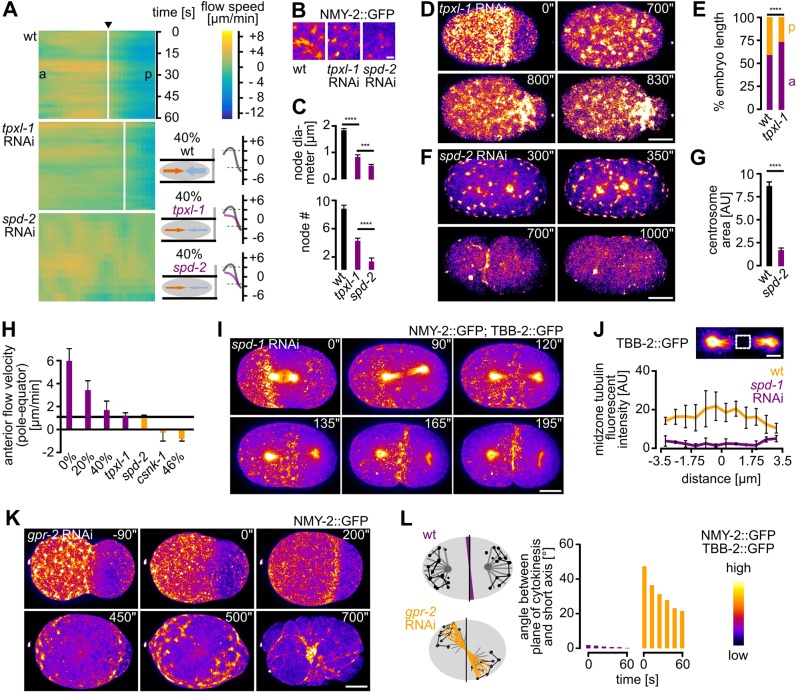
**Astral relaxation is crucial for cytokinetic cortical flow.** (A) Left: Heat map kymographs generated by PIV of NMY-2 particles along the long axis of 40% compressed one-cell wt, *tpxl-1* and *spd-2* RNAi embryos, respectively; a, anterior; p, posterior. Black arrow points to future furrow (*n*=5 each). Middle: Schematic highlighting the corresponding rotational cortical flow velocities. Right: Averaged velocities (over 60 s) along the anterior–posterior axis from the PIV analysis (right panels). Grey and red lines represent averaged velocities in wt and RNAi embryos, respectively (*n*=5 each). (B) Cropped stills from time-lapse microscopy of wt, *tpxl-1* and *spd-2* RNAi embryos expressing NMY-2::GFP; scale bar: 1 µm. The fluorescence intensity color code is shown at the bottom right of this figure. (C) Quantifications of NMY-2 node diameter (top) and number (bottom) of embryos used in A (*n*=5 each; ±s.d.). (D) Maximum projected stills from time-lapse microscopy of a representative *tpxl-1* RNAi embryo expressing NMY-2::GFP; scale bar: 10 µm. See also Movie 19. (E) Quantification of anterior (purple) and posterior (orange) domain lengths as a percentage of total embryo length in wt and *tpxl-1* RNAi embryos (*n*=5 each). (F) Representations as in panel D, however, for a representative *spd-2* RNAi embryo. See also Movie 20. (G) Centrosome areas quantified in wt and *spd-2* RNAi embryos. (*n*=5 each; ±s.d.). (H) Comparison of averaged velocities for pole-to-equator flow (shown here are flow velocities from the anterior pole only). The black line marks the velocity value above which furrowing still proceeds (*n*=5 each; ±s.d.). (I) Maximum projected stills from time-lapse microscopy of a representative *spd-1* RNAi embryo expressing NMY-2::GFP and TBB-2::GFP; scale bar: 10 µm. (J) Normalized fluorescence intensities (2.5×2.5 µm, boxed area) at the spindle midzone in wt (orange) and *spd-1* RNAi (purple) embryos (*n*=5 each). Scale bar: 2.5 µm. (K) Maximum projected stills from time-lapse microscopy of a representative *gpr-2* RNAi embryo expressing NMY-2::GFP; scale bar: 10 µm. (L) Left: Astral microtubule and polar node distribution as well as cleavage plane position for a wt and a *gpr-2* RNAi embryo. Right: Temporal dynamics of the angle between cleavage plane and the embryo's short axis for the two embryos depicted on the right. See also Movie 21. The color gradient at the bottom right shows the pseudo-coloring used to highlight NMY-2 or TBB-2 intensity in all panels of this figure.

First, we analyzed whether we can, indeed, observe spindle rotation upon unixial loading. Consistent with previous reports, we observed that particularly cortical spindle microtubules in the equatorial zone rotate (Fig. S6, Movie 17) and that this rotation occurs together with cortical NMY-2 (Fig. S6A, Movie 18). Second, we used RNAi of *tpxl-1* or *spd-2*, to shift the spindle to the posterior end or to remove astral microtubules, respectively (Decker et al., 2011; Lewellyn et al., 2011). For both *tpxl-1* and *spd-2* RNAi embryos, longitudinal flow (Fig. 6A; Fig. S6A), NMY-2 foci number and size were reduced (Fig. 6B,C). In *tpxl-1* RNAi embryos, cleavage furrows were significantly shifted to the posterior end, but cytokinesis occurred successfully (Fig. 6D,E; Movie 19). By contrast, in *spd-2* RNAi embryos (Fig. 6F), whose centrosomes are much smaller than those of wt embryos (Fig. 6G), cortical foci did not undergo flows and the cortex eventually ruptured (Movie 20). Although the phenotypes of *tpxl-1* and *spd-2* RNAi embryos have different mechanistic foundations, we calculated a lower limit of 1.2 µm/min compressive flow, where furrowing is still successful under mechanical load (Fig. 6H). Notably, this limit lies between the two conditions of uniaxial loading where cytokinesis always completes (40% compression, *n*>50) and where furrow formation is always unsuccessful (46% compression, *n*>25). To corroborate these data, we also analyzed the contribution of the central spindle. To directly test this, we used RNAi to inhibit gene expression of the microtubule bundling factor SPD-1 (PRC1 in humans). According to earlier work (Green et al., 2013), we found that, despite the lack of a spindle midzone, normal pole-to-equator flows are generated (Fig. 6I,J). Additionally, we also used RNAi to inhibit gene expression of the G-protein receptors GPR-1 and GRP-2, which are crucial for aster-positioned cytokinesis (Bringmann et al., 2007). We found that spindles were often not positioned along the long axis of the embryo (Fig. 6K). In these cases, cortical NMY-2 caps did not form at the poles of the embryo but around incorrectly positioned ends of astral microtubules (Fig. 6L). Accordingly, cortical flows were misoriented along the tilted spindle axis (Movie 21). Hence, polar relaxation seems crucial for the generation and correct polarization of cortical flow, which requires astral but not central spindle microtubules. Moreover, these data suggest that astral microtubules align cortical flow with the spindle and that their correct regulation is required for to induce rotational flow upon mechanical stress (summarized in Fig. S5).

### Cortical chirality and polarity are required for rotational flow polarization

**Fig. 7.**
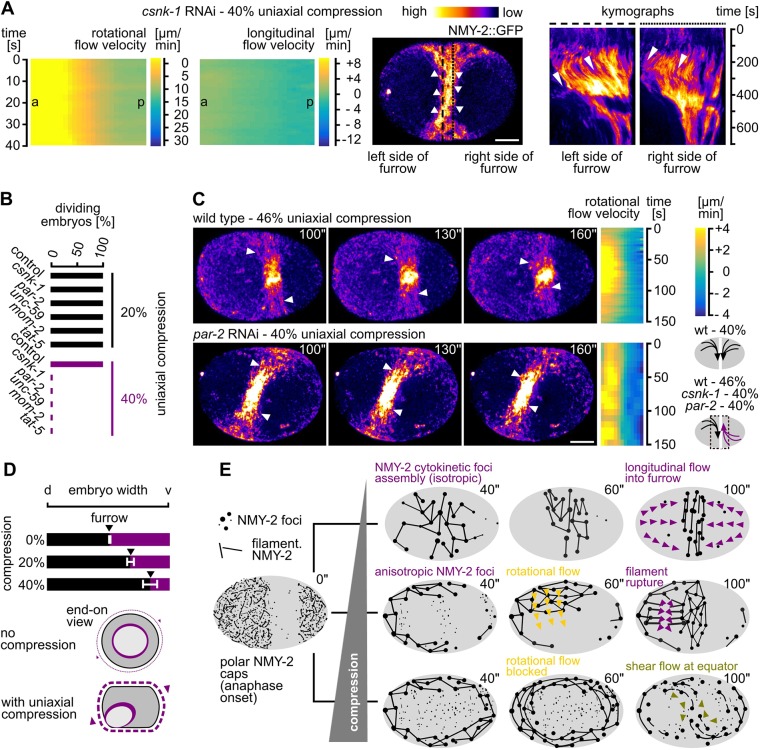
**Cortical polarity and chirality are required for mechanostable cytokinesis.** (A) Left: Two heat map kymographs generated by PIV of NMY-2 particles along the short and the long axis of one-cell *C*. *elegans* csnk-1 RNAi embryos. All embryos were imaged under 40% compression (*n*=5 each); a, anterior; p, posterior. Middle: Maximum projected still from time-lapse microscopy of a representative *csnk-1* RNAi embryo expressing NMY-2::GFP; white arrowheads indicate flow direction in the furrow region; scale bar: 10 µm. See also Movie 22. Right: Two kymographs generated along the dashed lines at the left and right boundaries of the furrow. Notice the opposite polarity of flow, indicated by arrowheads and the dissolution of the furrow after 400 s. (B) Quantification of successful first-cell division for the indicated RNAi treatments under 20% (black bars) and 40% (purple bars) compression (*n*=10 each). (C) Left: Maximum-projected stills from time-lapse microscopy of a representative wt (46% compressed) and *par-2* RNAi embryo (40% compressed) expressing NMY-2::GFP, and their accompanying heat maps generated through PIV of NMY-2 particle flow in the furrow region along the short axis (Scale bar: 10 µm; See also Movie 23). Right: Schematic depicting the polarization of the rotational flow in wt (top) and RNAi embryos (bottom). (D) Furrowing asymmetry quantified in wt embryos. Top: Average furrow position along the short axis is indicated by black arrowheads (*n*=5 each). Bottom: Schematic of a model, indicating how lack of rotational cortical flow influences furrow asymmetry. See text for details. (E) Model showing linearly organized cortical myosin dynamics under different conditions. Timing refers to onset of the formation of the polar cap (onset of anaphase) at 0 s. Left: Cortical NMY-2 distribution before the onset of cytokinesis. Top right: Linear and focal NMY-2 coalesce into an equatorial band in unstressed embryos through longitudinal pole-to-equator flow (purple arrowheads). Middle right: With increased loading, NMY-2 foci show anisotropic distribution at the onset of cytokinesis, for which most foci assemble laterally, on the bulged cortex. Subsequently, focal and linear NMY-2 show rotational flow (yellow arrowheads), and linearly organized NMY-2 ruptures, thereby, generating longitudinal flow (purple arrowheads). Bottom right: With high load, anisotropically distributed foci transform into a linearly organized network that shows shear flow (green arrowheads) in the equatorial region, leading to furrow disintegration.

Finally, we reasoned that regulators of cortical chirality contribute to rotational cortical flow polarization. To test this, we used RNAi to target gene expression of casein kinase 1γ (CSNK-1). In line with earlier observations (Singh and Pohl, 2014), we found that in *csnk-1* RNAi embryos, rotational cortical flows can switch their handedness across the equator and, concomitantly, leading to a strong reduction of compressive longitudinal flow (Fig. 7A, left; Fig. S7A). Importantly, the switch of rotational-flow handedness generated shear flow in the equatorial region, which lead to dissolution of the furrow under mechanical load (Fig. 7A, right, 40% compression; Movie 22). This phenotype was not restricted to *csnk-1* RNAi embryos but also occurred when components of the Wnt pathway that are required for cortical torque generation and chiral symmetry breaking (Pohl and Bao, 2010; Naganathan et al., 2014) were targeted by RNAi, i.e. *mom-2* (Fig. 7B).

Since the contractile ring forms by alignment of linearly organized cortical filaments through RhoA-dependent flow, the whole system also needs to be polarized along the direction of longitudinal compressive flow. Accordingly, we found that disruption of anterior–posterior polarity in *par-2* or *tat-5* RNAi embryos phenocopies the *csnk-1* and *mom-2* RNAi embryos, a lack of longitudinally polarized compressive flow and shear flow in the equatorial region (Fig. 7C; Fig. S7B,C, Movie 23). Importantly, we also observed shear flow in wt embryos that had been compressed 46% and did not divide (Fig. 7C; Movie 9). This suggests that, under these conditions, uniform rotational cortical polarization (observed in wt embryos compressed <40%) fails upon removal of factors responsible for cortical polarity and chirality or by excessive loading (Fig. 7C, bottom right). Next, we asked how furrowing itself is affected by uniaxial loading and tested whether factors known to be required for the intrinsic asymmetry of furrowing, such as septin-encoding *unc-59* (Maddox et al., 2007), are also involved (Fig. 7B). Similar to the requirement of genes involved in cortical polarity and chirality, we also found that *unc-59* RNAi embryos lack rotational cortical flow (100%, *n*=5) and fail to divide when compressed 40% (Fig. 7B).

Taken together, although factors involved in cortex polarity, chirality and asymmetry have not been found to be essential for cytokinesis in previous studies, they can become essential for cytokinesis under mechanical stress (Fig. 7B,C). Furthermore, since compression induces rotational flow and because all of the above RNAi embryos also show a loss of uniformly polarized rotational flow (Movies 22, 23), we measured the degree of asymmetric furrowing under increasing mechanical load. In accordance with the above findings, we found that furrowing becomes increasingly asymmetric with increased loading (Fig. 7D, top). These results, although correlative, strongly suggest that loading-induced rotational flows are involved in symmetry breaking during furrowing (Fig. 7D, bottom).

## DISCUSSION

Our data uncover a poorly characterized feature of cortical flow, its mechanosensitivity and its mechanostability through its ability to re-polarize from longitudinal to rotational (Fig. 7E). Moreover, we demonstrate that uniaxial compression is a straightforward experimental paradigm to systematically investigate the mechanobiology of cortical flow during asymmetric cell division. Importantly, this paradigm shows that the induction of rotational flow requires a correct mitotic spindle and that it depends on the magnitude of total mechanical stress. We also show that re-polarization of cortical flow is followed by anisotropic cortex rupture (Fig. 7E). Rupture can lead to equator-directed cortical flows during cytokinesis which result in cortical compression around the cell equator and furrowing. This seems to be one mechanism that can balance extrinsic and intrinsic forces during cytokinesis (Figs 2B and 3B). These results, therefore, extend previous work that identified longitudinal flows as contributors to contractile ring formation (Reymann et al., 2016; Khaliullin et al., 2018; Fig. S1C). In addition, our results reveal that, besides polarization of actin filaments through flow-alignment coupling (Reymann et al., 2016), cortical NMY-2 also shows flow-alignment coupling. However, by having much longer lifetimes, cortical NMY-2 shows higher flow velocities than F-actin and accumulates at the equator and in the midbody – unlike F-actin (Fig. 1). The recent thorough characterization of long, linearly organized NMY-2 stacks, whose lifetime is independent of the neighboring F-actin filaments (Hu et al., 2017) – together with our observation of different cortical flow profiles for NMY-2 and F-actin (lifeact) – strongly suggests that NMY-2 has roles during cell division that are separable from those of F-actin, in particular during final stages of contractile ring constriction and midbody formation (Fig. 1F,G). Moreover, the proposed attractive interactions between linearly organized NMY-2 (Hu et al., 2017) might also explain why we observed NMY-2 flows with longer duration and range than F-actin flows.

Our experiments (Fig. 6) also extend previous work showing that the whole embryo rotates (Schonegg et al., 2014; Bolková and Lanctôt, 2016) and that astral microtubule regulators are involved in cortical flow (Mangal et al., 2018). By dissecting the differential contribution of the astral and the midzone pathway, we uncover an essential role for the astral pathway in cytokinesis mechanostability. Increasing mechanical stress alone or in combination with a delay of aster separation through *tpxl-1* RNAi (Fig. 6D,E; Lewellyn et al., 2011) induces a posterior shift of the furrow (Fig. 2B). This suggests that the interaction between astral microtubules and cortical force generators is, itself, mechanosensitive. Additionally, reducing centrosome size and, concomitantly, astral microtubule density through *spd-2* RNAi (Fig. 6G; Decker et al., 2011) shows that strong loss of astral microtubules leads to a reduction of cytokinetic NMY-2 foci (Fig. 6F) and to a reduction of furrow-directed flow. Thus, by altering the position or number of astral microtubules – without manipulating global NMY-2 levels or NMY-2 activators – it is possible to identify a lower threshold for astral microtubule-induced flow beyond which cytokinesis cannot proceed under mechanical stress (Fig. 6H).

Moreover, it also seems likely that anisotropies in spindle organization and spindle-cortex contacts pattern local cortex activation and, thereby, flow polarization (Fig. S6, Movie 18). Thus, it is tempting to speculate that strong cortical flows are restricted to cytokinesis since the cortex only here shows sufficient excitability (Bement et al., 2015) and the spindle can induce patterned activation/inactivation of RhoA that will generate polarized flows. In part, the coupling of astral microtubule dynamics to cortical dynamics might explain why we observed cortical behaviors that suggest a substantial elastic contribution to material behavior in addition to the previously characterized viscoelastic behavior. To corroborate the range of elastic behavior, future experiments using timed and oscillatory compression will be highly informative. Remarkably, an apparatus introduced by Douglas Marsland (Marsland, 1950) allowed to test the effect of isobaric compression on cytokinesis in sea urchin embryos. It revealed that a critical pressure of ∼400 bar at room temperature is required to induce furrow regression. In accordance with our view that the cortex of the entire embryo contributes to cytokinesis through cortical flow, Marsland suggested that a gel-like system can only drive cytokinesis if it is “forming a continuous and fairly extensive system throughout the cell”.

In addition, we demonstrate that several pathways, all of which have specific, non-redundant functions outside cytokinesis, fulfill essential roles for rotational cortical flow and furrow stability when cells are mechanically stressed (Figs 4–7). These pathways include the PAR and the Wnt pathways, which are known for their role in specifying the anteroposterior and the left–right body axes, respectively. Only for the PAR pathway a connection to cortical dynamics during cytokinesis is known (Jordan et al., 2016). Remarkably, interference with any of these pathways resulted in similar mechanical stress-dependent failure of cytokinesis, a loss of uniform rotational cortical flow polarization, which lead to shear flow and dissolution of the contractile ring (Fig. 6). This suggests that correct anteroposterior cortical polarization and yet-to-be-identified aspects of cortical polarity that relate to cortical torque generation, become essential for furrowing under mechanical stress. Additionally, we found that correct regulation of actomyosin, which is required for intrinsically asymmetric furrowing, is also essential for cytokinesis mechanostability. These data support earlier findings upon which it was argued that, when the intrinsic asymmetry is disrupted, cytokinesis becomes sensitive to partial inhibition of contractility (Maddox et al., 2007).

Although the data we present here are correlative in many aspects, nevertheless, they suggest that cortical rotation and cytokinesis mechanostability are intricately linked and rely on factors that are, presumably, required for symmetry breaking during cytokinesis. Moreover, our data also suggest that generation of cortical torque depends on linear organization of cortical NMY-2 (Fig. 5). However, increased cortical torque alone is not sufficient for cytokinesis to proceed normally under load. Under these conditions, remodeling of linear cortical structures seems crucial for the re-distribution of contractile cortical material towards the cleavage furrow by longitudinal flow and assembly of a contractile equatorial ring. Taken together, our findings show that Ray Rappaport's notion that the cytokinesis machinery is “overbuilt, inefficient, never-failed, and repaired by simple measures” (Srivastava et al., 2016) might only be appropriate for unstressed cells. However, apparently redundant factors can become essential under mechanical stress.

## MATERIALS AND METHODS

### Worm strains, worm maintenance and RNA interference

Integrated *C. elegans* strains expressing lifeact-fusion proteins expressed from *pie-1* promoters have been described elsewhere (Pohl and Bao, 2010; Pohl et al., 2012). Strains JJ1473 (zuIs45), LP162 (*nmy-2(cp13)*, and RW10223 (itIs37; stIs10226) were provided by the Caenorhabditis Genetics Center (CGC). A strain expressing a sensor for active RohA, mgSi5[cb-UNC-119 (+) GFP::ANI-1(AH+PH)II; unc-64(e246)III], was kindly provided by Michael Glotzer (Tse et al., 2012). Strains were maintained under standard conditions (Brenner, 1974). RNAi was performed through feeding, using clones from commercially available libraries (Fraser et al., 2000; Rual et al., 2004).

### Microscopy and laser ablation

Embryo preparation and mounting has been described elsewhere (Pohl and Bao, 2010; Dutta et al., 2015). Mounting was modified by using differently sized polystyrene (15 µm, 20 µm, 25 µm; Polysciences, Hirschberg, Germany) and polymethylmethacrylate spheres (12 µm and 13.5 µm, PolyAn, Berlin, Germany). Microscopy was performed with a VisiScope spinning disk confocal microscope system (Visitron Systems, Puchheim, Germany) based on a Leica DMI6000B inverted microscope, a Yokogawa CSU X1 scan head, and a Hamamatsu ImagEM EM-CCD. All acquisitions were performed at 21–23°C using a Leica HC PL APO 63×/1.4-0.6 oil objective. Cell cortex ablations were performed using a pulsed 355 nm UV laser mounted on the same microscope. One ablation cycle was performed per acquisition with a residence time per pixel of 3.5 ms. Acquisitions pre and post ablation were performed at 200-ms intervals.

### Particle image velocimetry

We measured the cortical flows in the developing embryos by using a post-processing technique called particle image velocimetry (PIV) analysis. A PIV analysis computes the displacement vector between two different time points based on their cross-correlation. For this, we used a Matlab custom code of the cortical flows in *C. elegans* embryos based on the two-dimensional code from PIVlab (Thielicke and Stamhuis, 2014; Dutta et al., 2015). Complete details of our implementation and validation of the PIV method in early *C. elegans* embryos are discussed in Dutta et al., 2015. Here, we used NMY-2::GFP as an *in situ* marker to capture cortical dynamics by measuring magnitude and direction of the cortical flow. We pre-processed the images by setting values larger than the median plus two times the standard deviation to the maximum intensity. The biological timing for the start point of our analysis is the onset of cytokinetic foci formation, with at least three NMY-2 foci with a radius ≥1 µm being present. This timing correlates to onset of anaphase. Analysis was started 1 min afterwards, in a window comprising 30 sequential frames spanning 60 s of embryo development until the start of visible furrowing. Given a pair of sequential images, we sought to obtain a maximum-likelihood estimate (MLE) for the displacement vector. To this end, we used a two-step linear operation. First, a coarse or predictor displacement vector was found by using normalized cross-correlation, operating on equally spaced overlapping windows of 64×64 pixels. Cross correlation was calculated in the Fourier space by using the convolution theorem. This was repeated until a vector file for every window has been calculated. In the subsequent corrector step, the coarse displacement predictions were used to offset the sub-windows of 32×32 pixels by an integer pixel amount, and the cross-correlation procedure was repeated. In this iteration, however, the search radius was reduced to a size in the order of the error made by the predictor approximation. The calculated velocities were then thin-plate spline interpolated to obtain a smoothly varying and continuous displacement vector field.

### Quantification and kymograph representation of flow profiles – statistics

The flow profile for each time point was projected on the long axis of the embryo by dividing the whole vector profile of the embryo into 13 bins and taking a mean along the short axis. A time course profile or kymograph was obtained by averaging bin velocities for five embryos under each condition. For visualization, heat maps were generated after applying cubic interpolation by using a custom MATLAB script. Variability between embryos for each condition was estimated by calculating the standard error of the mean and plotting this for the entire vector matrix.

### Measurements – statistics

NMY-2 and TBB-2 signal intensities, NMY-2 foci number and size, NMY-2 filament contraction rate of linearly organized NMY-2, cortical residence times of NMY-2 and lifeact, NMY-2 outward flow velocities, spindle microtubule angles, as well as furrow asymmetry and anterior–posterior domain sizes were manually measured in ImageJ using the built-in toolset (Schindelin et al., 2012). Cortical residence times were measured from traces in kymographs or by tracking cortical structures in sequential frames of high-resolution time-lapse series. Longitudinal flow-range was measured in the anterior domain by extracting continuous tracks from PIV data that showed velocities of >0.5 µm/min and normalizing them to embryo length. Cleavage success was manually quantified by inspecting time-lapse microscopy data.

For shape parameter quantification of embryos *in utero* (Fig. 3A), the embryo perimeter was segmented using a custom MATLAB script by applying a median filter and thresholding. Circularity was defined as 4π (area/perimeter^2^).

Calculation of curvature to quantify blebbing (Fig. S4A) was performed by segmentation of cell boundaries using a custom MATLAB script. For each time point, the boundary at the anterior end of the embryo was divided into 400 equidistant points. A circle was fit for each boundary point by using this point and two boundary points that were four points away. The local curvature was defined as reciprocal of the radius of this fitted circle.

To establish whether the *C. elegans* embryo follows Laplace's law (Fig. 2A), sideview projections of embryos were obtained by using a custom MATLAB script. Projected images were denoised (Wiener filter) and the bounary of the embryo was segmented by adaptive thresholding. For each point on the boundary, a circle was fitted on three points with a spacing of 30 points. Curvature was defined as the inverse of the radius of the fitted circle. Contact angles were measured on the basis of segmented boundaries.

If not stated otherwise, time points refer to time point 0 s as onset of polar cap formation, which precedes cortical foci formation during cytokinesis (equivalent to anaphase onset) by ∼40 s.

All above measurements represent at least five biological replicates if not noted otherwise and data are represented with standard deviation (+s.d.) as error bars.

## Supplementary Material

Supplementary information
