# Supplementary material for: Mechanical stress induces a scalable switch in cortical flow polarization during cytokinesis
Source: J Cell Sci. 2019 Oct 9;132(19):jcs231357. doi: 10.1242/jcs.231357 (PMC6803361; doi:10.1242/jcs.231357)
Supplement: Supplementary information [file joces-132-231357-s1.pdf]

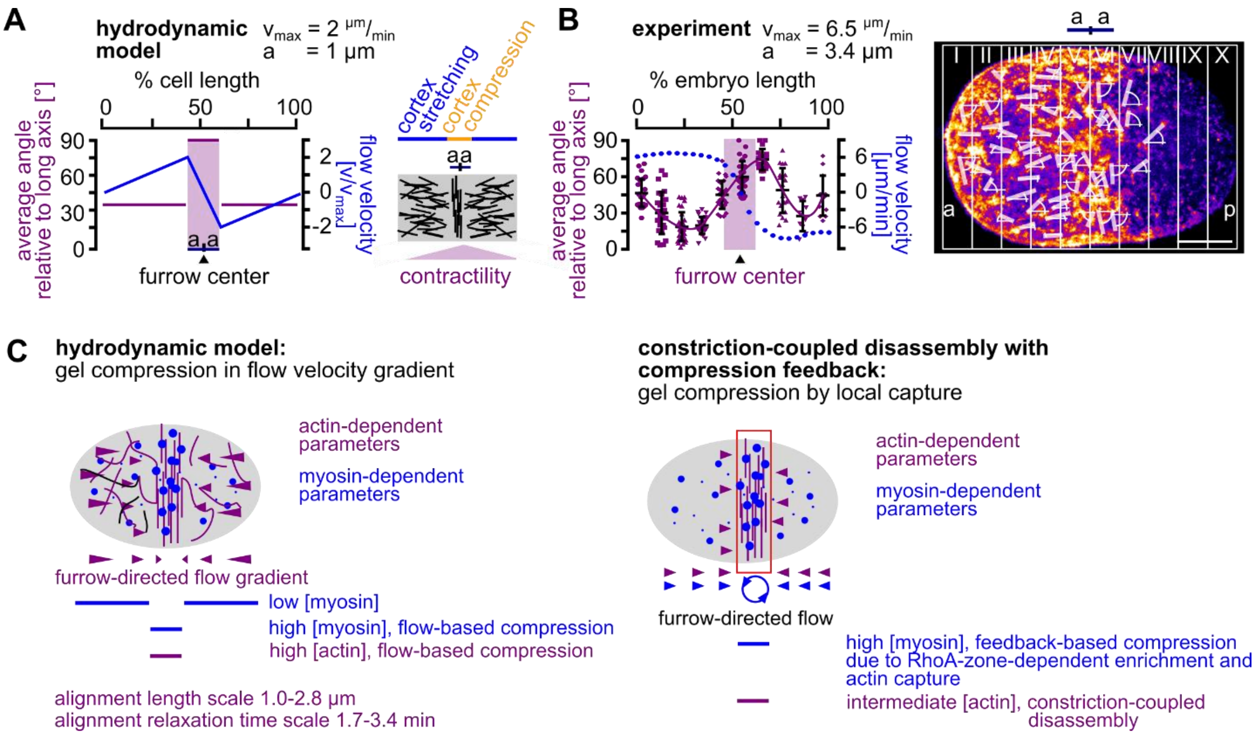

Fig. S1: Comparison of NMY-2 dynamics to a hydrodynamic model of furrowing and to a constriction-coupled disassembly and compression feedback model. (A) Quantification of NMY-2 linear orientation. Left: Distribution of order parameter and flow velocity for a cylindrical system undergoing cytokinesis (see cartoon according to Salbreux et al., 2009). (B) Left: Measured angle and flow velocities along the a-p axis (n = 5). Right: Representative embryo with angles of linearly organized NMY-2 relative to the a-p-axis. (C) Summary of the recently proposed models on contractile ring formation through a cortical flow gradient and self-alignment of actin (Reymann et al., 2016, left) and by equatorial RhoA zone-dependent local compression of the actomyosin cortex and disassembly by myosin (Khaliullin et al., 2018). Major differences are that the gel compression model does not require a defined RhoA zone and postulates lack of myosin-dependent, active alignment. In contrast, the capture-compression model requires myosin activity in the equatorial region to capture adjacent actomyosin cortex. Compression in the equatorial zone will lead to disassembly, explaining the reduced amount of actin relative to myosin in the contractile ring.

Journal of Cell Science • Supplementary information

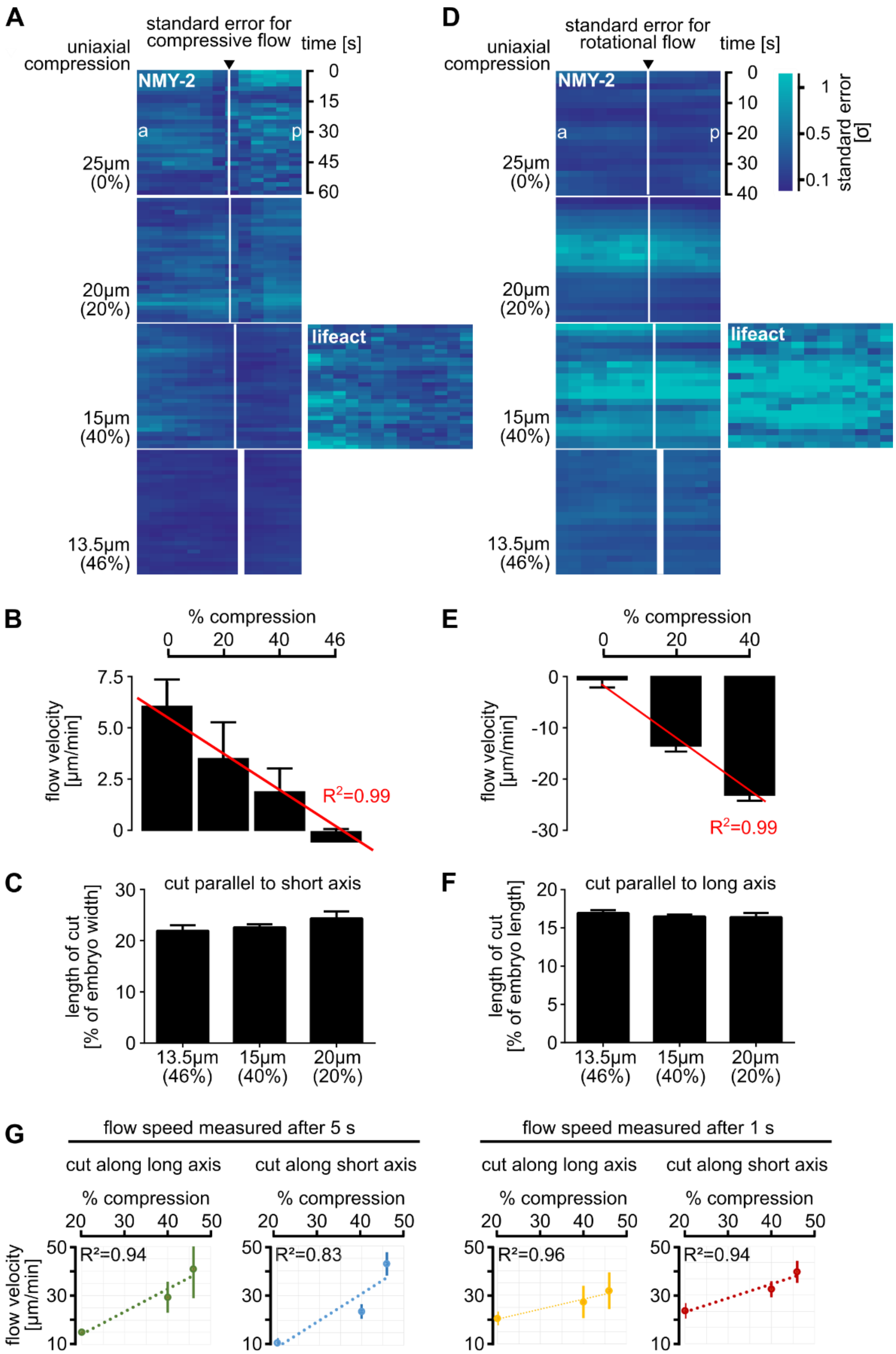

Journal of Cell Science • Supplementary information

Fig. S2: Statistics for orthogonal flows and cortical ablations in wild type embryos. (A) Standard errors for each pixel in the heat map kymographs of Fig. 2B, 2C. (B) Scaling of compressive flows with increasing compression. Average flow velocities for the anterior domain are shown with standard deviations. (C) Length of wounds after cortical laser ablation along the short axis (n = 5). (D) Standard errors for each pixel in the heat map kymographs of Fig. 3B, 3C. (E) Scaling of rotational flows with increasing compression. Average flow velocities are shown with standard deviations. (F) Length of wounds after cortical laser ablation along the long axis (n = 5). (G) Linear regressions for outward cortical flow velocities after UV laser cortex ablations of embryos under different compression, see also Fig. 2D, 3G.

Journal of Cell Science • Supplementary information

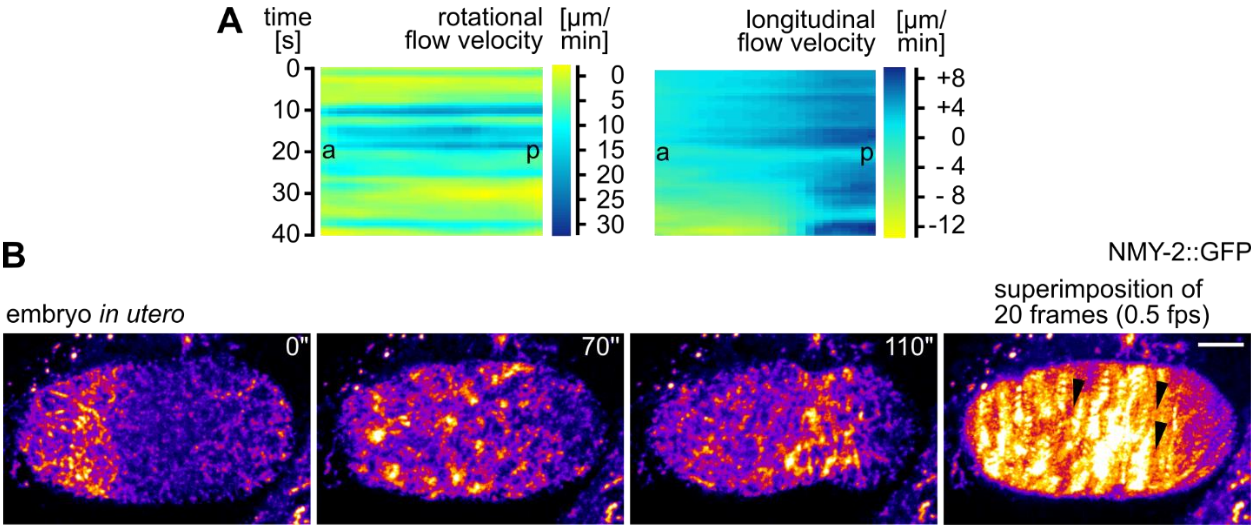

Fig. S3: Cortex rotation *in utero*. (A) Representative PIV analysis of an embryo *in utero* during cytokinesis. Heat map kymographs generated by PIV of NMY-2 particles along the short and the long axis, respectively. It turned out that due to variability of loading and positioning of embryos in the uterus, a representation of averages from ensembles is not feasible. (B) Maximum projected still from time lapse microscopy of a representative embryo in the uterus expressing NMY-2::GFP; black arrowheads indicate flow direction; scale bar = 10 µm. See also Movie 4.

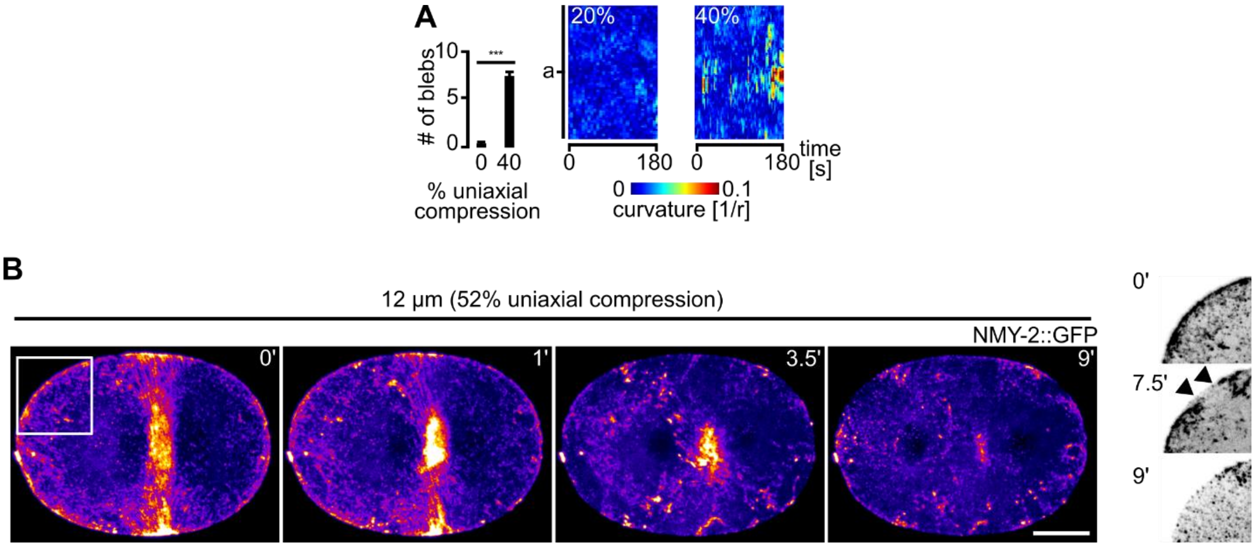

Fig. S4: Polar blebbing is a response to uniaxial loading and increased loading can lead to cortex rupture. (A) Left: Quantification of the number of blebs in uncompressed and 40% compressed WT embryos over 60 s. Right: Quantification of curvature changes. Two representative curvature kymographs for a 20% and a 40% compressed embryo are shown. See materials and methods for details. (B) Cortex rupture for 52% compression. Representative projections from time-lapse microscopy are shown; scale bar = 10 µm. The right pictures show the boxed area of the leftmost still annotated with arrowheads and inverted to illustrate cortex rupture. See also Movie 10.

Journal of Cell Science • Supplementary information

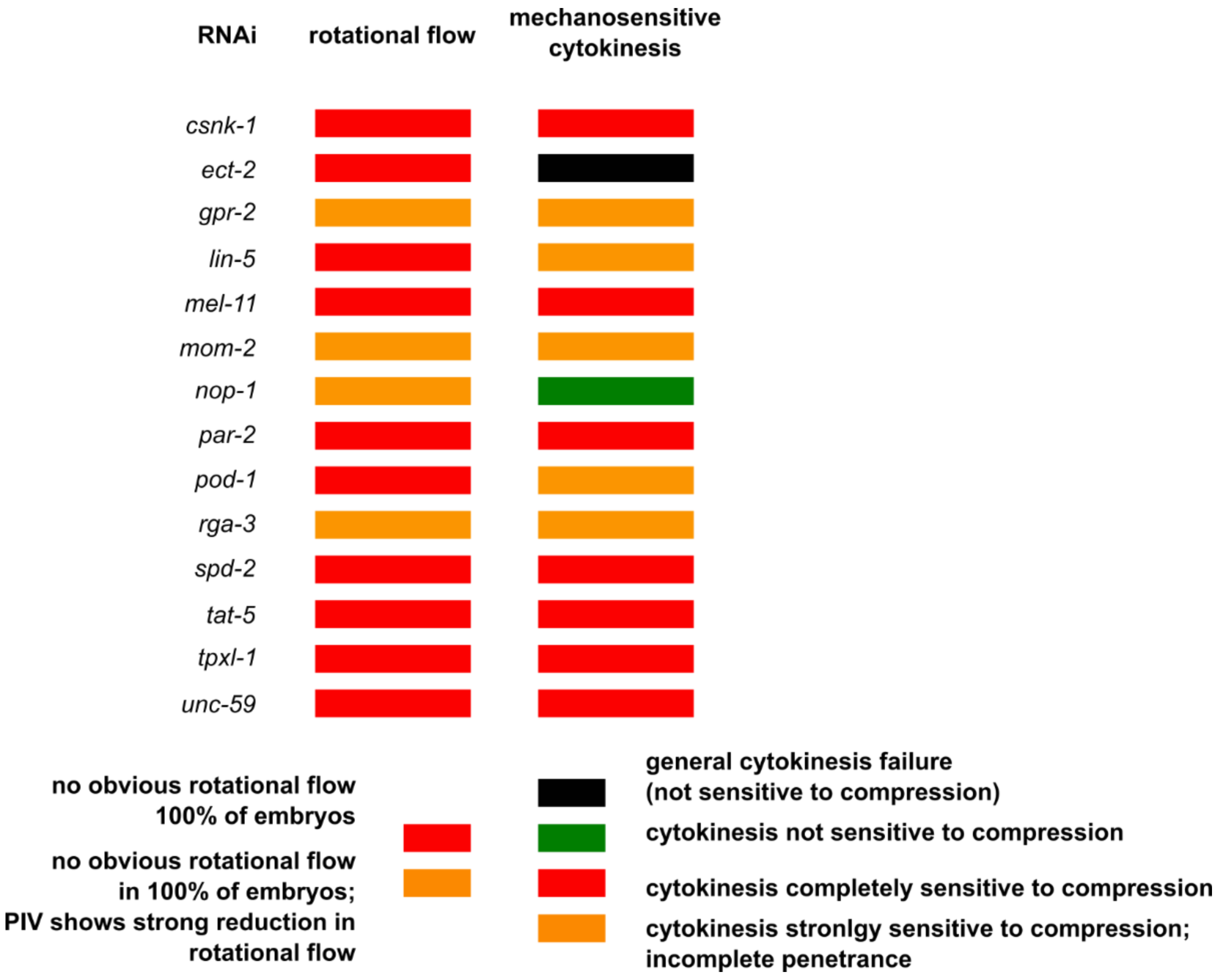

Fig. S5: List of genes tested for their role in cortical rotation. All genes tested in our small-scale, targeted screen are listed here with a summary of the phenotypes observed. Wherever quantifications have been possible by PIV, they have been included in the main figures. Quantification of cytokinesis mechanosensitive failure have been performed manually, n ≥ 5 for each RNAi.

Journal of Cell Science • Supplementary information

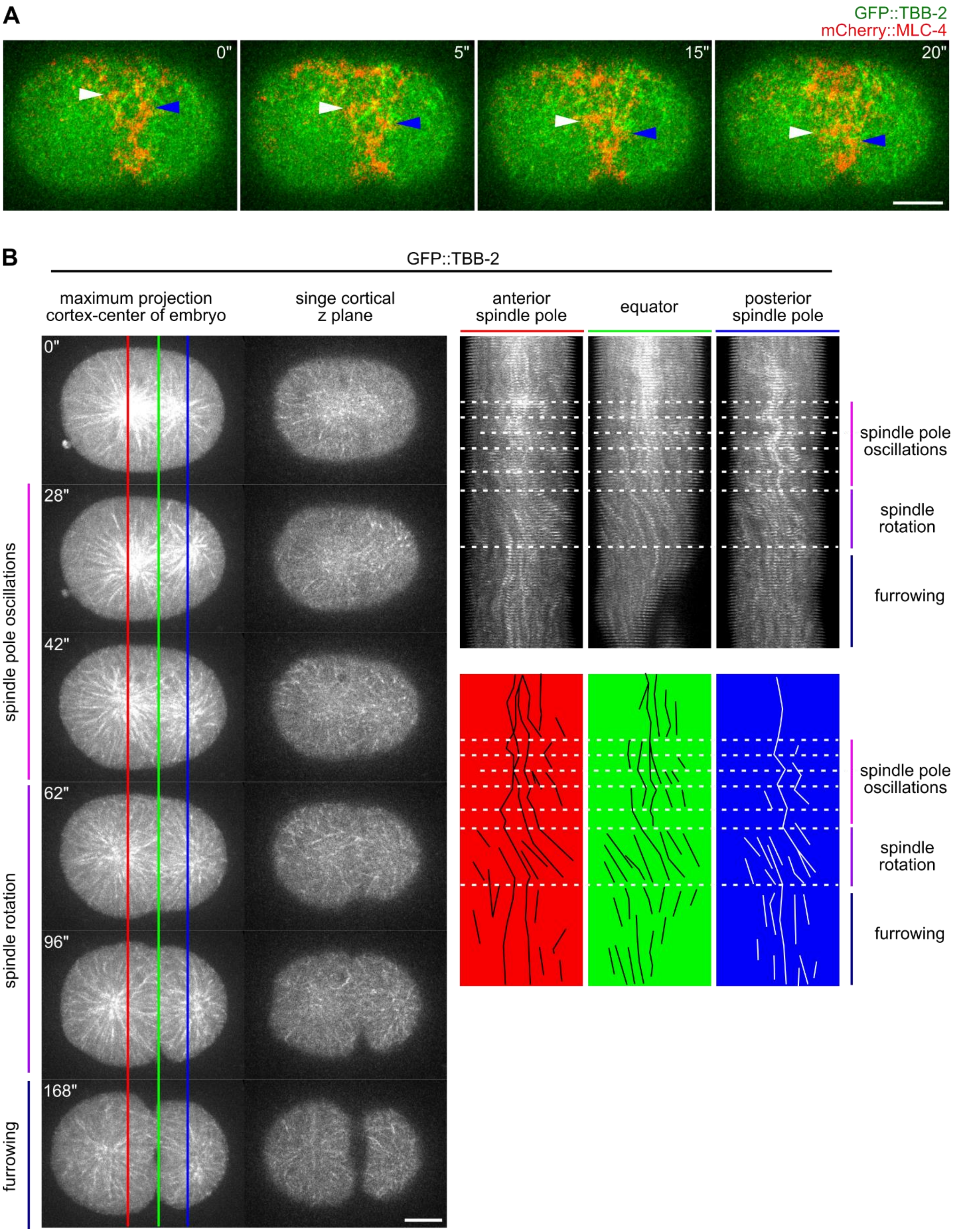

Journal of Cell Science • Supplementary information

Fig. S6: Rotation of the spindle. (A) Single cortical plane snapshots of a representative time lapse recording of a 40% compressed embryo (see also Movie 18) showing rotation of cortical microtubules (TBB-2) together with cortical non-muscle myosin II (MLC-4). White and blue arrowheads mark rotationally translocating cortical material. The embryo was 20% compressed. Scale bar = 10 µm. (B) Rotation of spindle microtubules is most apparent in the cortex. Left: Maximum projection snapshots of the upper half and matching topmost cortical planes from a representative time-lapse recording. Coloured lines mark the lines used to generate the corresponding kymographs on the right. Right: Kymographs (top) and traced trajectories of individual cortical microtubules (bottom). Any deviation of tracks from a straight line indicates oscillation or rotation. Oscillations of spindle poles result in opposite direction turning of anterior *vs.* posterior tracks, spindle rotation shows a uniform turning of tracks from anterior to posterior. Note that anterior microtubules seem to rotate slightly stronger than posterior. Scale bar = 10 µm.

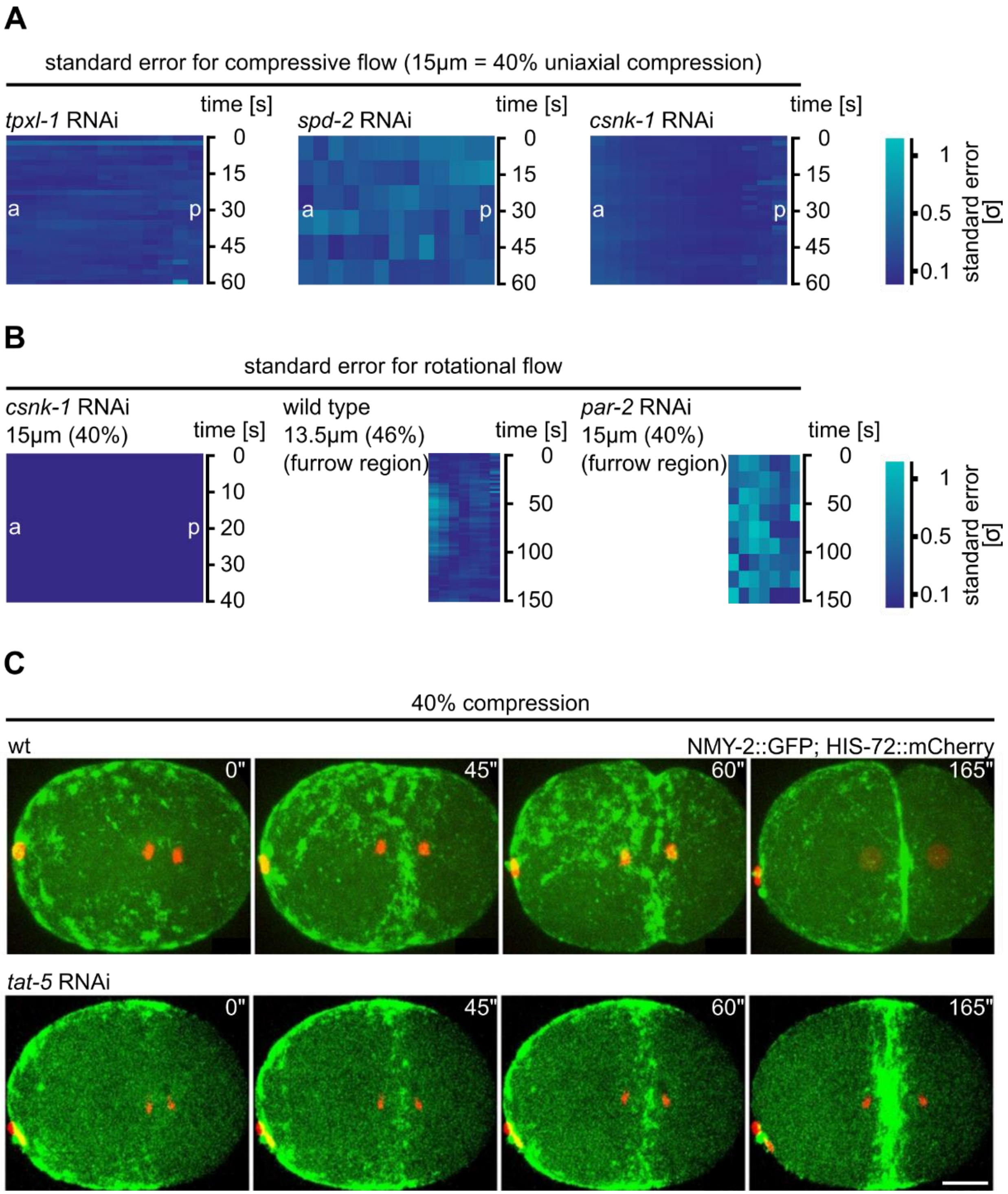

Fig. S7: Statistics for cortical flows in RNAi embryos and cytokinesis failure in *tat-5* RNAi embryos. (A) Standard errors for each pixel in the compressive flow heat map kymographs of Fig. 6A, 7A. (B) Standard errors for each pixel in the rotational flow heat map kymographs of Fig. 7A, 7C. (C) Maximum projected stills from time lapse microscopy of NMY-2 dynamics in representative wild type and a *tat-5* RNAi embryos. Scale bar = 10 µm.

Journal of Cell Science • Supplementary information

Movies

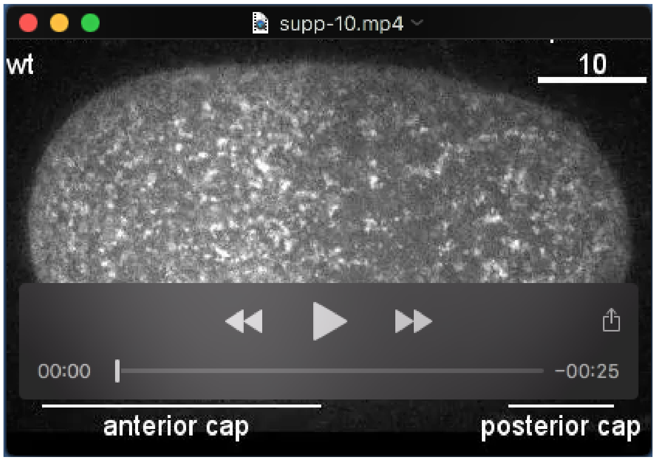

Movie 1

A time lapse series of a Z-projected unstressed wild type embryo expressing NMY-2::GFP. White bars indicate NMY-2 domains in anterior and posterior, red circles mark cortical nodes, red arrowheads point to cortical filaments and compressive flow, red squares mark particles streaming towards to equator; scale bar = 10 µm.

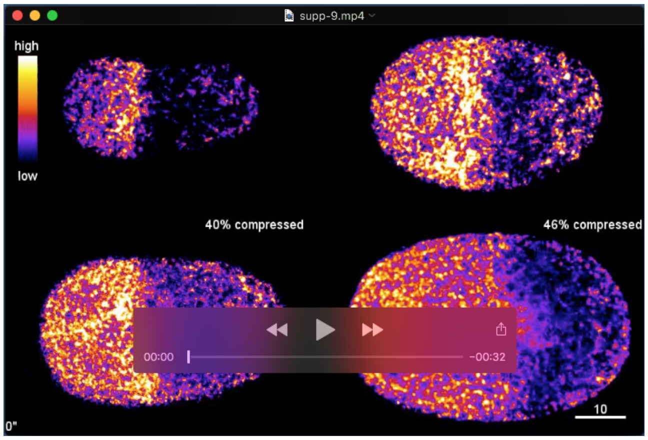

Movie 2

A concatenated time lapse series of Z-projected wild type embryos expressing NMY-2::GFP ranging from unstressed to 46% uniaxial compression; scale bar = 10 µm.

Journal of Cell Science • Supplementary information

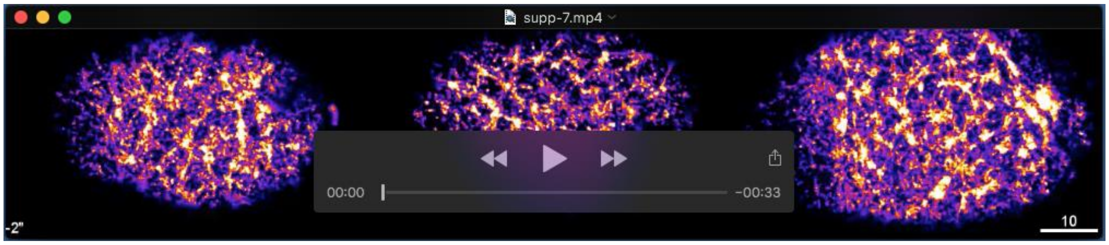

Movie 3

A concatenated time lapse series of Z-projected wild type embryos expressing NMY-2::GFP where UV-laser cutting was performed along the short axis of the embryo; scale bar = 10 µm.

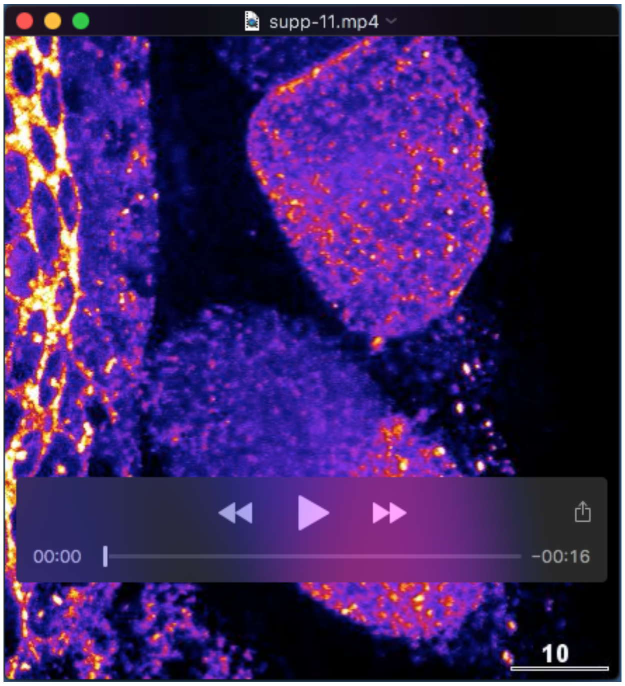

Movie 4

A time lapse series of a Z-projected parts of the gonad and two embryos *in utero* expressing NMY-2::GFP. White circles indicate rotating cortical foci. Note that the embryo adjacent to the embryo undergoing the first division is oriented dorso-ventrally, indicating that embryos are actually compressed orthogonal to the axis of observation; scale bar = 10 µm.

Journal of Cell Science • Supplementary information

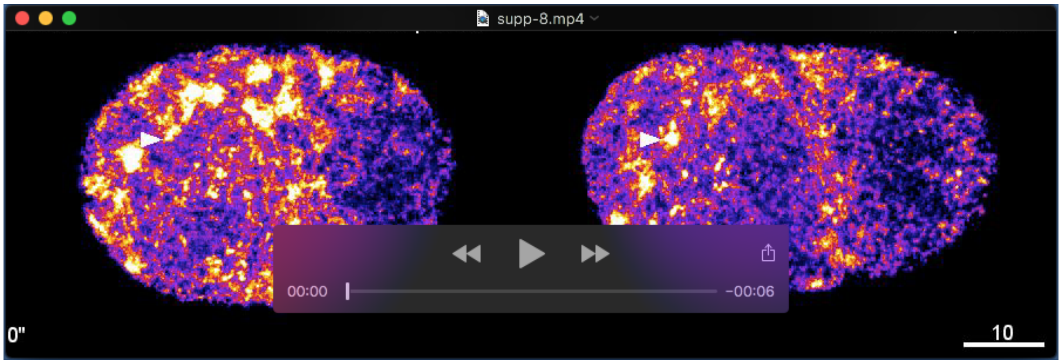

Movie 5

A concatenated time series of Z-projected wild type embryos expressing NMY-2::GFP under differential compression, exhibiting different rotational velocities indicated by white arrowheads; scale bar = 10 µm.

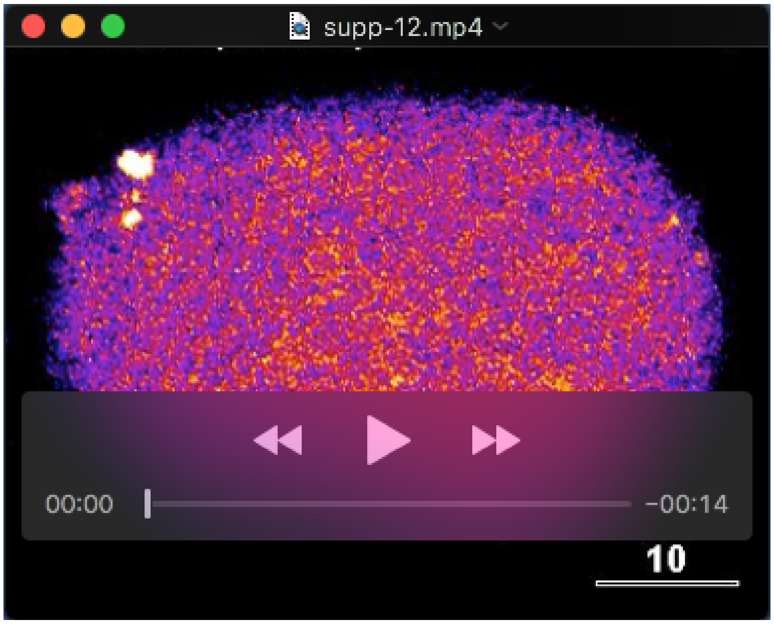

Movie 6

A time lapse series of a Z-projected wild type embryo expressing GFP::ANI-1(AH+PH), a sensor for active RhoA, under 40% compression; scale bar = 10 µm.

Journal of Cell Science • Supplementary information

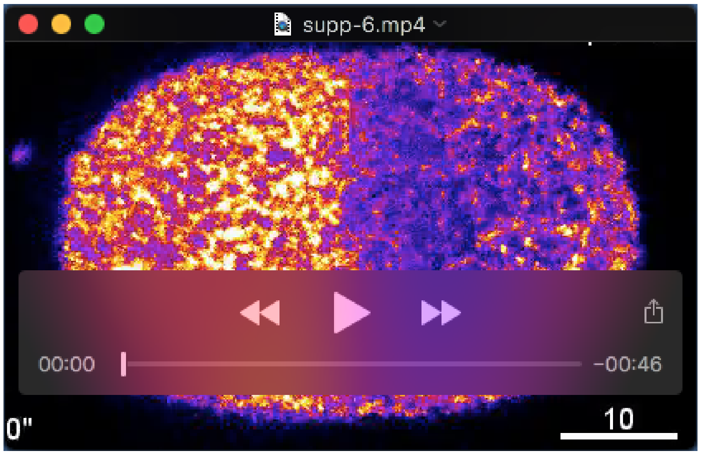

Movie 7

A time lapse series of a Z-projected wild type embryo expressing NMY-2::GFP under 40% compression. NMY-2::GFP nodes are encircled in white, white arrowheads point to rupturing filaments and also indicate polar blebbing; scale bar = 10 µm.

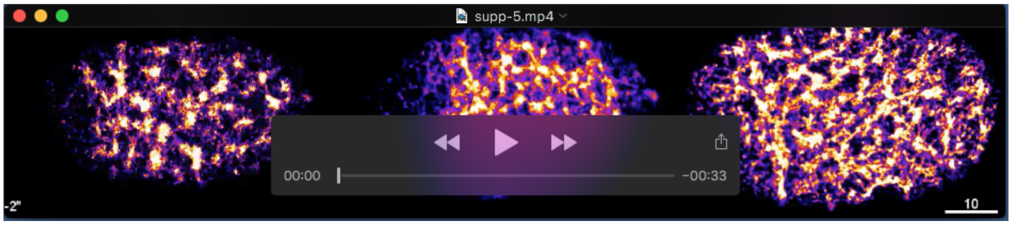

Movie 8

A concatenated time lapse series of Z-projected wild type embryos expressing NMY-2::GFP where UV laser cutting was performed along the long axis of the embryo; scale bar = 10 µm.

Journal of Cell Science • Supplementary information

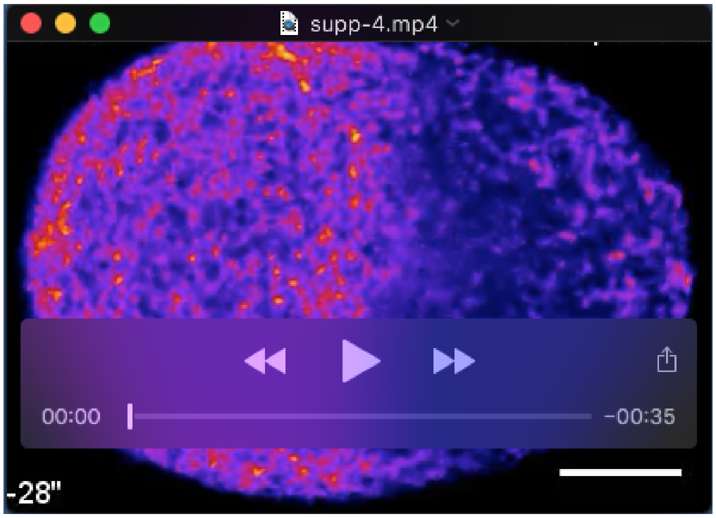

Movie 9

A time lapse series of a Z-projected wild type embryo expressing NMY-2::GFP under 46% compression. White circles indicate traces of individual NMY-2::GFP particles; scale bar = 10 µm.

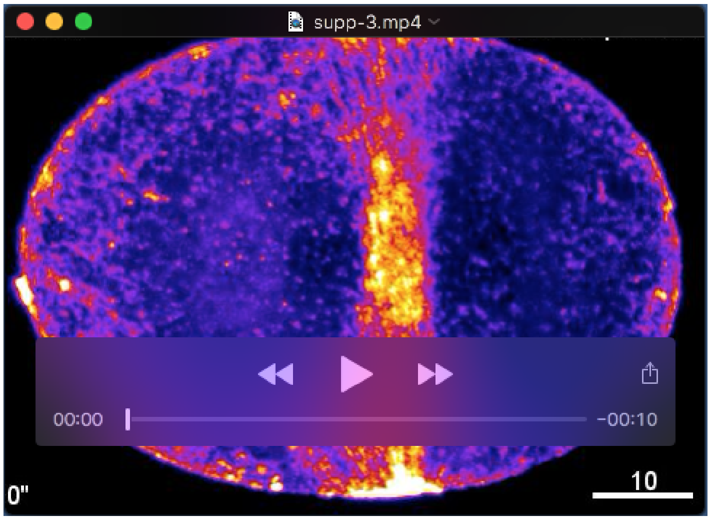

Movie 10

A time lapse series of a Z-projected wild type embryo expressing NMY-2::GFP under 52% compression; scale bar = 10 µm.

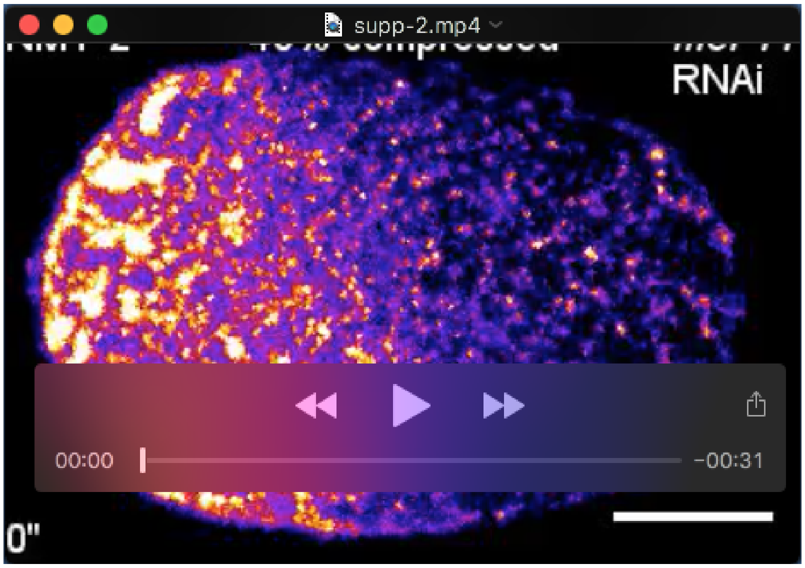

Movie 11

A time lapse series of a Z-projected *mel-11* RNAi embryo expressing NMY-2::GFP under 40% compression; scale bar = 10 µm.

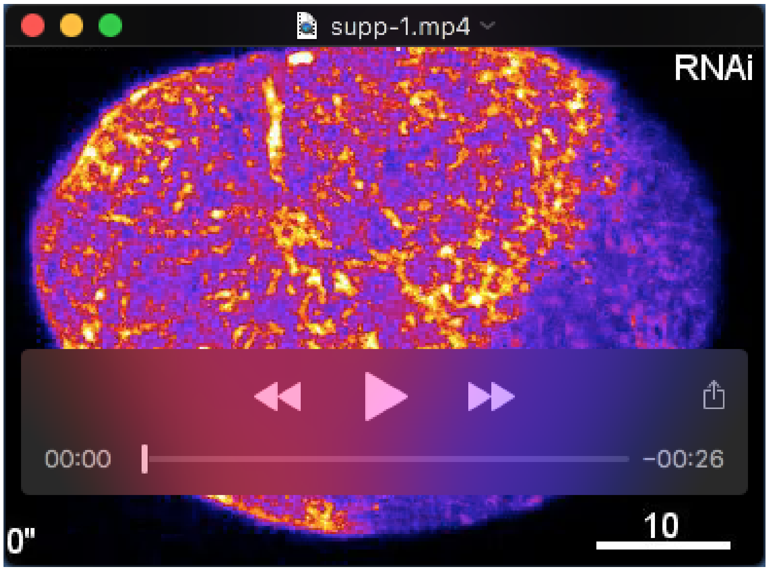

Movie 12

A time lapse series of a Z-projected *lin-5* RNAi embryo expressing NMY-2::GFP under 40% compression; scale bar = 10 µm.

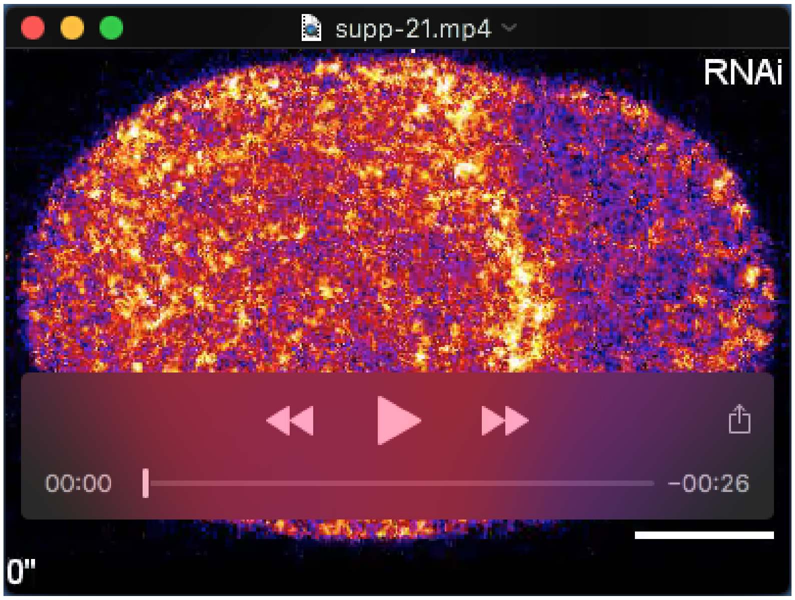

Movie 13

A time lapse series of a Z-projected *ect-2* RNAi embryo expressing NMY-2::GFP under 40% compression; scale bar = 10 µm.

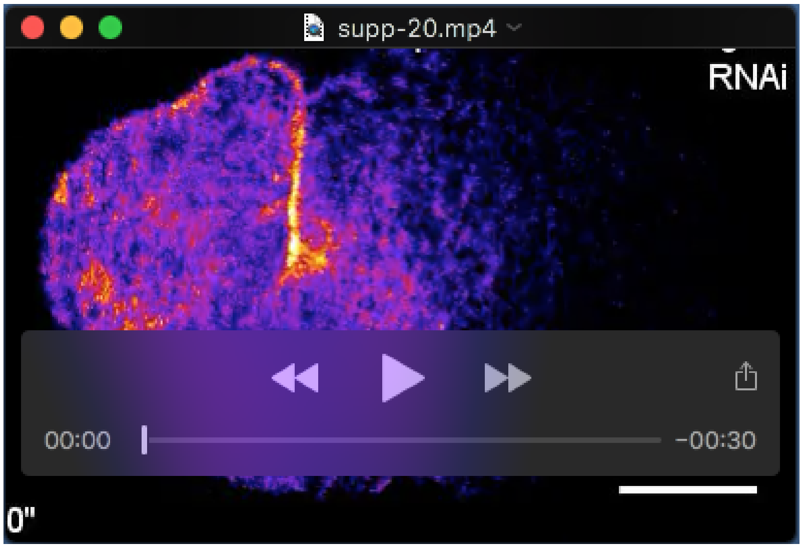

Movie 14

A time lapse series of a Z-projected *rga-3* RNAi embryo expressing NMY-2::GFP under 40% compression; scale bar = 10 µm.

Journal of Cell Science • Supplementary information

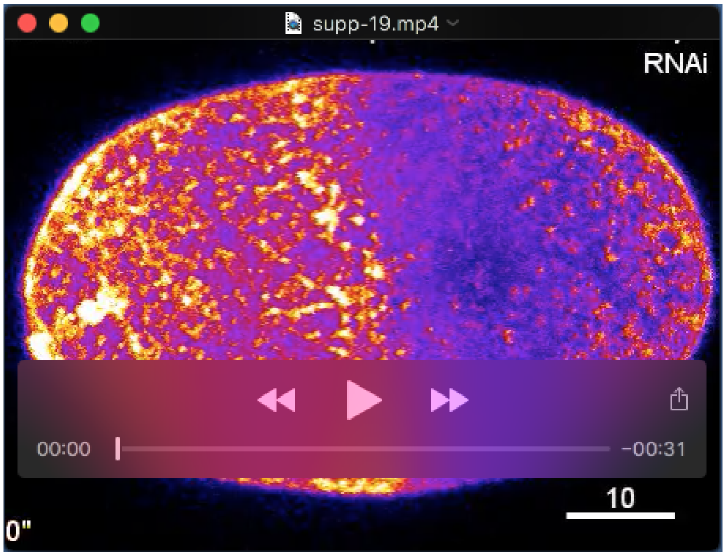

Movie 15

A time lapse series of a Z-projected *nop-1* RNAi embryo expressing NMY-2::GFP under 40% compression; scale bar = 10 µm.

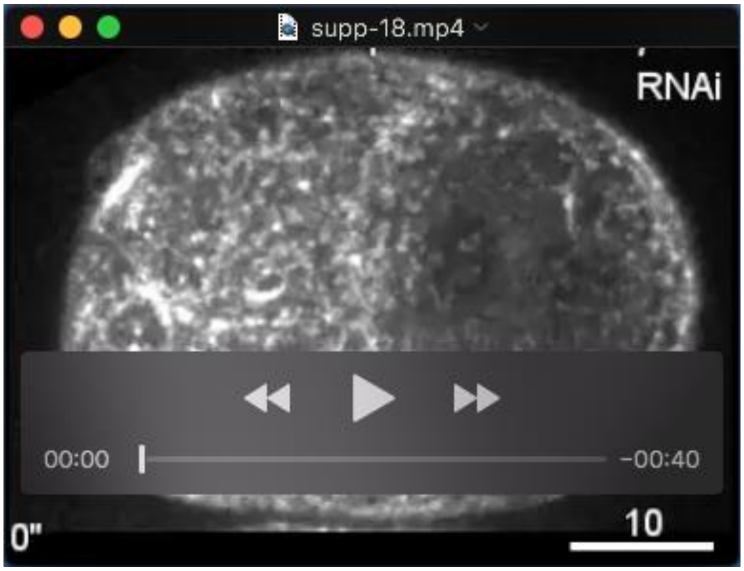

Movie 16

A time lapse series of a Z-projected *pod-1* RNAi embryo expressing NMY-2::GFP under 40% compression. Red arrowheads point to contractile NMY-2 structures; scale bars are 10 µm (first part) and 2.5 µm (second part).

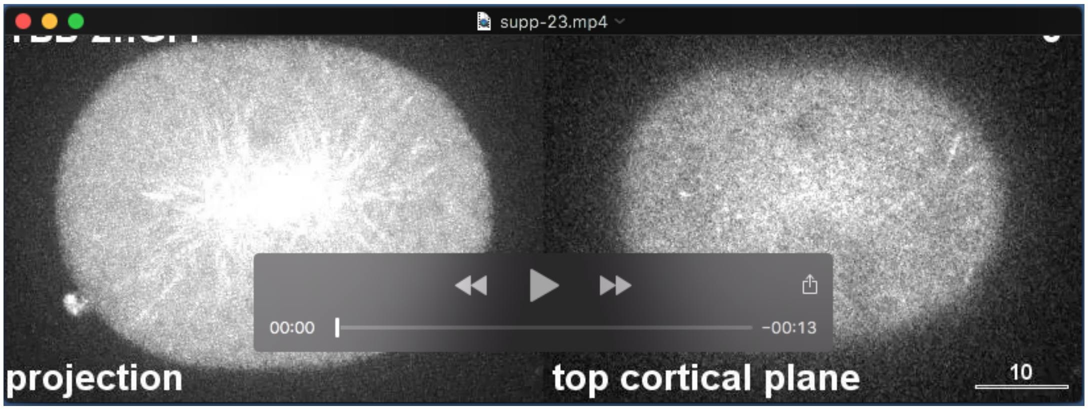

Movie 17

A time lapse series of a embryo expressing GFP::TBB-2 under 40% compression; scale bar = 10 µm. Shown are the z-projected top half of the embryo and the top cortical plane. White circles show tracking of cortical microtubules that undergo rotation.

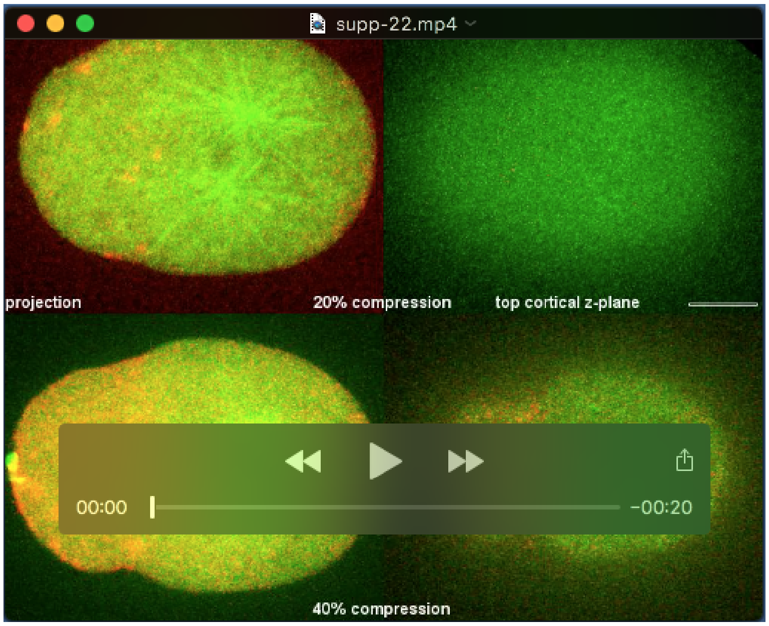

Movie 18

Time lapse series of embryos expressing GFP::TBB-2 and mCherry::MLC-4 under 20% (top) and 40% compression (bottom); scale bar = 10 µm. Shown are the z-projected top half of embryos and the top cortical planes.

Journal of Cell Science • Supplementary information

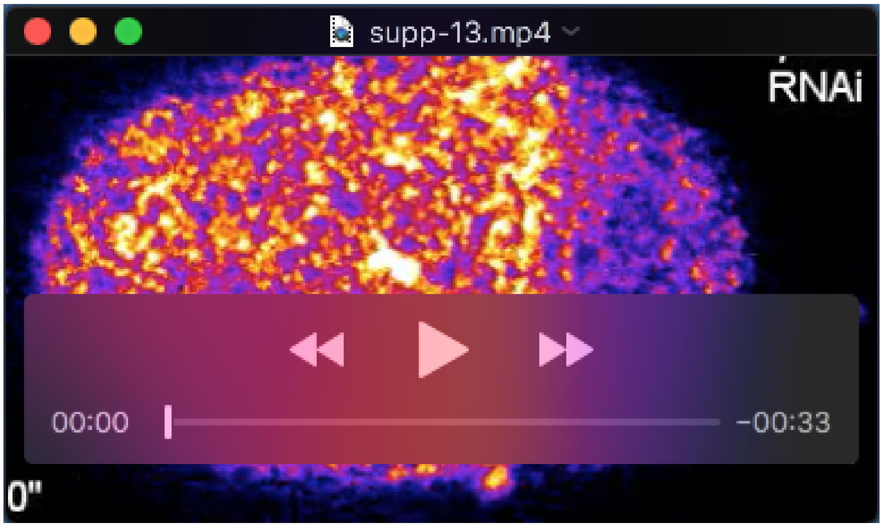

Movie19

A time lapse series of a Z-projected *tpxl-1* RNAi embryo expressing NMY-2::GFP under 40% compression; note the strongly delayed formation of cytokinetic NMY-2 foci and their long lifetime compared to wt embryos; scale bar = 10 µm.

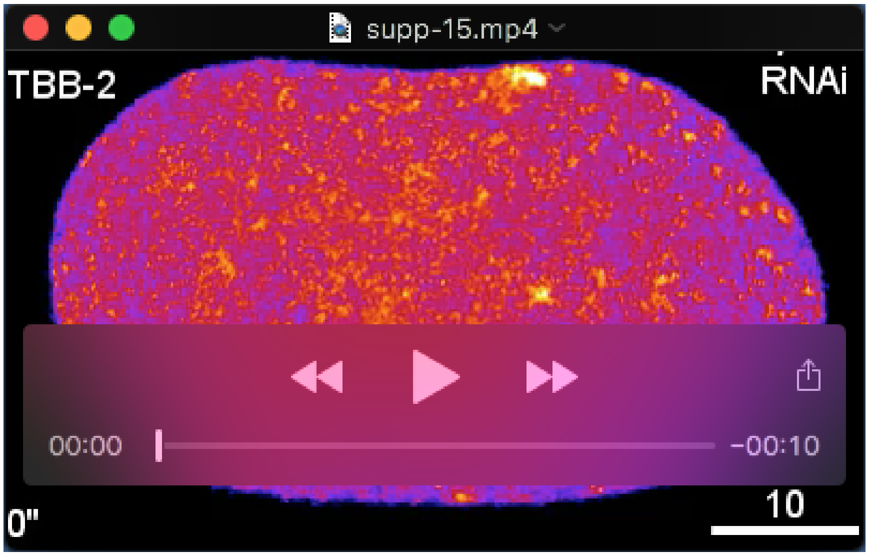

Movie 20

A time lapse series of a Z-projected *spd-2* RNAi embryo expressing GFP::TBB-2 and NMY-2::GFP under 40% compression; scale bar = 10 µm.

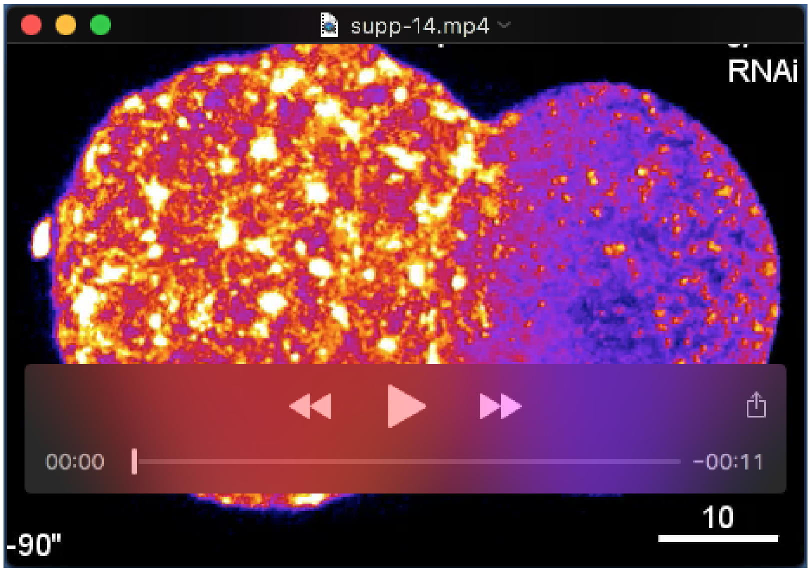

Movie 21

A time lapse series of a Z-projected *gpr-2* RNAi embryo expressing GFP::TBB-2 and NMY-2::GFP under 40% compression; scale bar = 10 µm.

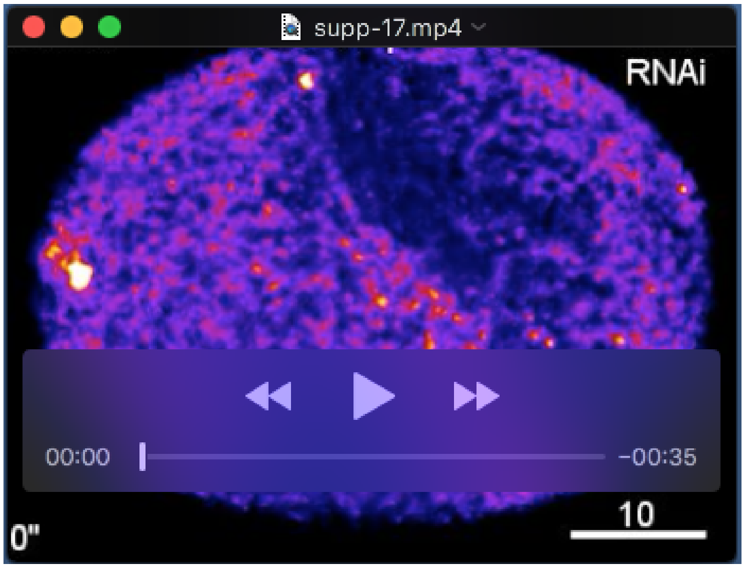

Movie 22

A time lapse series of a Z-projected *csnk-1* RNAi embryo expressing NMY-2::GFP under 40% compression; scale bar = 10 µm.

Journal of Cell Science • Supplementary information

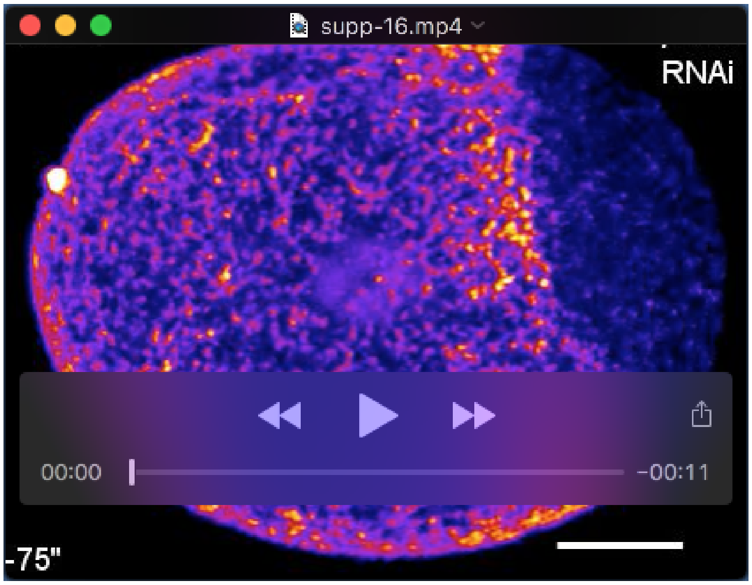

Movie 23

A time lapse series of a Z-projected *par-2* RNAi embryo expressing NMY-2::GFP under 40% compression. White circles indicate traces of individual NMY-2::GFP particles; scale bar = 10 µm.

Journal of Cell Science • Supplementary information
